# Ultrastructural Characteristics and Synaptic Connectivity of Photoreceptors in the Simplex Retina of Little Skate (*Leucoraja erinacea*)

**DOI:** 10.1523/ENEURO.0226-23.2023

**Published:** 2023-10-25

**Authors:** Laura Magaña-Hernández, Abhiniti S. Wagh, Jessamyn G. Fathi, Julio E. Robles, Beatriz Rubio, Yaqoub Yusuf, Erin E. Rose, Daniel E. Brown, Priscilla E. Perry, Elizabeth Hamada, Ivan A. Anastassov

**Affiliations:** Department of Biology, San Francisco State University, San Francisco, CA 94132

**Keywords:** little skate, photoreceptors, retina, rods, SB-3DEM, simplex

## Abstract

The retinas of the vast majority of vertebrate species are termed “duplex,” that is, they contain both rod and cone photoreceptor neurons in different ratios. The retina of little skate (*Leucoraja erinacea*) is a rarity among vertebrates because it contains only a single photoreceptor cell type and is thus “simplex.” This unique retina provides us with an important comparative model and an exciting opportunity to study retinal circuitry within the context of a visual system with a single photoreceptor cell type. What is perhaps even more intriguing is the fact that the *Leucoraja* retina is able use that single photoreceptor cell type to function under both scotopic and photopic ranges of illumination. Although some ultrastructural characteristics of skate photoreceptors have been examined previously, leading to a general description of them as “rods” largely based on outer segment (OS) morphology and rhodopsin expression, a detailed study of the fine anatomy of the entire cell and its synaptic connectivity is still lacking. To address this gap in knowledge, we performed serial block-face electron microscopy imaging and examined the structure of skate photoreceptors and their postsynaptic partners. We find that skate photoreceptors exhibit unusual ultrastructural characteristics that are either common to rods or cones in other vertebrates (e.g., outer segment architecture, synaptic ribbon number, terminal extensions), or are somewhere in between those of a typical vertebrate rod or cone (e.g., number of invaginating contacts, clustering of multiple ribbons over a single synaptic invagination). We suggest that some of the ultrastructural characteristics we observe may play a role in the ability of the skate retina to function across scotopic and photopic ranges of illumination. Our findings have the potential to reveal as yet undescribed principles of vertebrate retinal design.

## Significance Statement

The vast majority of vertebrate retinas are duplex and have mixed rod-cone populations of photoreceptors. The processing of visual information in a duplex retina is separated between rods and cones, which mediate function under scotopic and photopic lighting conditions, respectively. However, the cartilaginous fish little skate (*Leucoraja erinacea*) has a simplex retina, comprised solely of one photoreceptor cell type. Skate photoreceptors are unusual because they have the ability to retain function over a full range of illumination. We know little about the ultrastructural anatomy of the skate retina, and we hypothesize that functional plasticity can be traced back to morphologic adaptations at the level of photoreceptors and downstream circuitry, thus illuminating new pathways for the processing of visual information among vertebrates.

## Introduction

The little skate (*Leucoraja erinacea*) is a member of the elasmobranchii subclass of cartilaginous fishes and has been a consequential model system for studies of fin and limb development (Barry and Crow, 2017; Marlétaz et al., 2023), skeleton formation (Criswell et al., 2017), electroreception (Bellono et al., 2018) and even the evolution of walking on land (Jung et al., 2018). The skate visual system has received a lot less attention, but appears to be no less fascinating. For example, the neural retina of this animal goes against the trend of the vast majority of other vertebrate retinas and appears to be comprised of only a single photoreceptor cell type (Dowling and Ripps, 1990; Ripps and Dowling, 1990). Earlier studies classified skate photoreceptors as “rods” based on their outer segment (OS) morphology (Szamier and Ripps, 1983), cloning and phylogeny of rod opsin (J. O’Brien et al., 1997), spectral profile of the photoreceptor chromophore (Ripps and Brin, 1977; Sun and Ripps, 1992), and photoreceptor sensitivity (Carter Cornwall et al., 1989). However, key aspects of the physiology of skate photoreceptors, such as their remarkable ability to adapt to photopic light (Carter Cornwall et al., 1989; Dowling and Ripps, 1972), as well as their unusual structural characteristics when one looks beyond the outer segment, as we describe in the present study, belie the “rod” characterization and we shall refer to them here simply as “photoreceptors.”

Retinas with a single photoreceptor cell type are rare. Instead, a mixed rod-cone photoreceptor retina is a lot more typical among vertebrates, where rods and cones are found in different ratios (Baden et al., 2020), and where rods mediate scotopic (dim light) vision, while cones mediate photopic (daylight) vision (Rodieck, 1998). Thus, the little skate has provided us with a unique opportunity to study a single photoreceptor cell type retina (Hu et al., 2021), unencumbered by some of the artifacts of genetic manipulation in rodents (Nikonov et al., 2005; Williams et al., 2005). Perhaps even more surprising, however, is the fact that the skate retina can function under both scotopic and photopic light conditions with a monotypically pure photoreceptor system (Carter Cornwall et al., 1989). A number of elegant classical studies by Dowling, Ripps, and Cornwall have shown that under scotopic conditions, skate photoreceptors can function at the theoretical threshold of sensitivity and detect single photons (Dowling and Ripps, 1972; Green et al., 1975; Carter Cornwall et al., 1989). But, after a relatively brief period of light-adaptation, they speed up their kinetics, lower their sensitivity, and expand their functional capabilities to photopic levels of illumination (Dowling and Ripps, 1971; Carter Cornwall et al., 1989). Furthermore, the downstream components of the skate retinal circuitry can also adapt from scotopic to photopic conditions (and back) and continue to transmit the visual message (Dowling and Ripps, 1971; Qian and Ripps, 1992). How this functional plasticity in skate photoreceptors and the downstream circuitry happens is still not entirely understood and it is surprising that we also know very little about the ultrastructural anatomy and connectivity of skate retinal neurons.

To the best of our knowledge, only three studies have examined any aspects of the ultrastructure of neurons in the skate retina in appreciable detail. Szamier and Ripps (Szamier and Ripps, 1983) used conventional electron microscopy to examine the juvenile skate retina and describe the disk shedding properties of skate photoreceptors, showing that what appeared to be morphologically cone-like cells in younger animals were in fact immature rod-like cells. In a separate study, Malchow and colleagues (Malchow et al., 1990) examined the ultrastructural and functional properties of two types of skate horizontal cells and showed that they are physiologically and anatomically distinct. Finally, Ripps and Chappell (Ripps and Chappell, 1991) examined the ultrastructural changes to skate photoreceptor terminals and neurotransmitter vesicles during prolonged depolarization and showed that synaptic terminals (STs) undergo a large increase in the extent of plasma membrane, as would be predicted with large exocytosis events of neurotransmitter vesicles.

Fortunately, recent advances in 3D ultrastructural imaging have provided us with an opportunity to resolve and examine fine details of skate retina anatomy and to begin assembling a connectome of this unique visual system. In the present study, we used serial block-face scanning electron microscopy (SB-3DEM), a cutting-edge method for anatomic reconstruction, to examine the ultrastructure of skate photoreceptors and the postsynaptic processes invaginating into photoreceptor terminals. We show that skate photoreceptors display structural elements that are commonly found in other vertebrate rods, mixed with elements that are either more typical of vertebrate cones, or likely unique to skate photoreceptors. We suggest that elements of skate photoreceptors’ unique architecture may be connected to the ability of the skate visual system to function across scotopic and photopic ranges of illumination. For example, our observation that different numbers of ribbons can be found across different photoreceptor terminals might suggest different levels of synaptic transmission and excitation of postsynaptic partners across lighting conditions. Taken together, our findings have the potential to significantly expand our understanding of the vertebrate visual system and reveal undescribed principles of vertebrate retinal design.

## Materials and Methods

### Animals

All animal procedures followed IACUC approved animal protocols at the Marine Biological Laboratory and San Francisco State University. Wild caught adult and in-house bred female and male juvenile little and winter skate animals (*L. erinacea* and *Leucoraja ocellata*) were obtained from the Marine Biological Laboratory Marine Resources Center. They were kept in a recirculating seawater system at 12–13°C. Circulating seawater was subjected to continuous physical, biological and chemical filtration. Animals were kept under a 12/12 h light/dark cycle and fed finely chopped frozen squid and mysids once a day. Both species (*L. erinacea* and *L. ocellata)* were used in this study, as they are closely related, frequently co-habit in the wild, and extensive studies have shown no anatomic or physiological differences between the retinas of either species (Dowling and Ripps, 1990; Ripps and Dowling, 1990). Animals were monitored daily for health and signs of general distress and all studies were performed following animal euthanasia. Before euthanasia, animals were anesthetized with sodium bicarbonate-buffered 0.02% tricaine methanesulfonate (Syndel) until unresponsive, followed by fast cervical transection and pithing. This method of euthanasia is consistent with the American Veterinary Medical Association (AVMA) Guidelines on Euthanasia.

### Tissue preparation

Retinal tissue was harvested following euthanasia from the eyes of three adult skates (two female and one male). Eyes were enucleated under ambient illumination; the cornea and lens were removed and the vitreous drained. Photoreceptors should be considered light-adapted under these conditions and likely in their more extended morphology, bringing them closer to the retinal pigment epithelium (RPE). The retina was left attached to the choroid and cartilaginous sclera to protect it from structural damage and aid in subsequent sectioning. Samples derive from the horizontal visual streak area of the retina situated directly above the tapetum lucidum, a reflective strip embedded in the choroid (Jinson et al., 2018; Lisney et al., 2012). The tapetum was used as a reference point during dissections. More specifically, samples derive from a few square millimeters taken out of presumed area of the central horizontal streak, immediately dorsal to the optic nerve head. The resulting eyecups were immediately fixed with 4% paraformaldehyde + 2.5% glutaraldehyde in 0.1 m cacodylate buffer (pH 7.2) for 5–7 d at 4°C. Fixed samples were shipped on ice to the 3DEM Ultrastructural Imaging and Computation Core at the Cleveland Clinic Lerner Research Institute (Cleveland, OH), where tissue was subjected to postfixation with OsO_4_, graded dehydration with ethanol, en bloc staining with uranyl acetate, and infiltration and embedding in epoxy resin.

### Imaging

Imaging of samples and collection of raw data were performed at the Cleveland Clinic 3DEM Ultrastructural Imaging and Computation Core. Large volume 3D electron microscopy was performed on retinal pieces embedded in epoxy resin using the serial block-face scanning electron microscopy method (SB-3DEM). A Teneo Volumescope system (Thermo Fisher Scientific) and a Zeiss Sigma VP system (Carl Zeiss Microscopy GmbH) equipped with a Gatan 3View in-chamber ultramicrotome stage, were used to image the different samples. Samples were sectioned and imaged in the cross-sectional orientation, which allowed for the visualization of all retinal layers and cell types. The datasets used and analyzed in the present study are from a region of interest (ROI) in the outer plexiform layer (OPL; P2R9 volume) and from a full cross-section of the retina (HVMS volume). The region of interest dataset had a width and height of 27.6 μm and a depth of 21.5 μm; voxel size was 4.5 × 4.5 × 70 nm (*xyz*). The full cross-section dataset had a width of 88 μm, a height of 304 μm and a depth of 22 μm; section thickness is 0.075 μm; voxel size was 10 × 10 × 75 nm (*xyz*).

### Data analysis and statistical procedures

Segmentation, 3D reconstructions, surface area and volume measurements were obtained with Reconstruct software (Fiala, 2005). All other quantitative measurements were obtained with Amira software (Thermo Fisher Scientific). Quantitative and statistical analyses were completed with Prism software (GraphPad Software). Two-tailed unpaired *t* tests with and without Welch’s correction were used for comparisons of photoreceptor OS and IS area, volume and length. OS and IS diameter parameter comparisons were done with a two-tailed Mann–Whitney test. *p*-values and replicates are listed in the figure legends and main text. Least squares Gaussian fit was used for curves shown in histogram data describing observations of ribbon and telodendrion numbers.

## Results

### The simplex retina of *L. erinacea* contains only a single photoreceptor cell type

**Figure 1.**
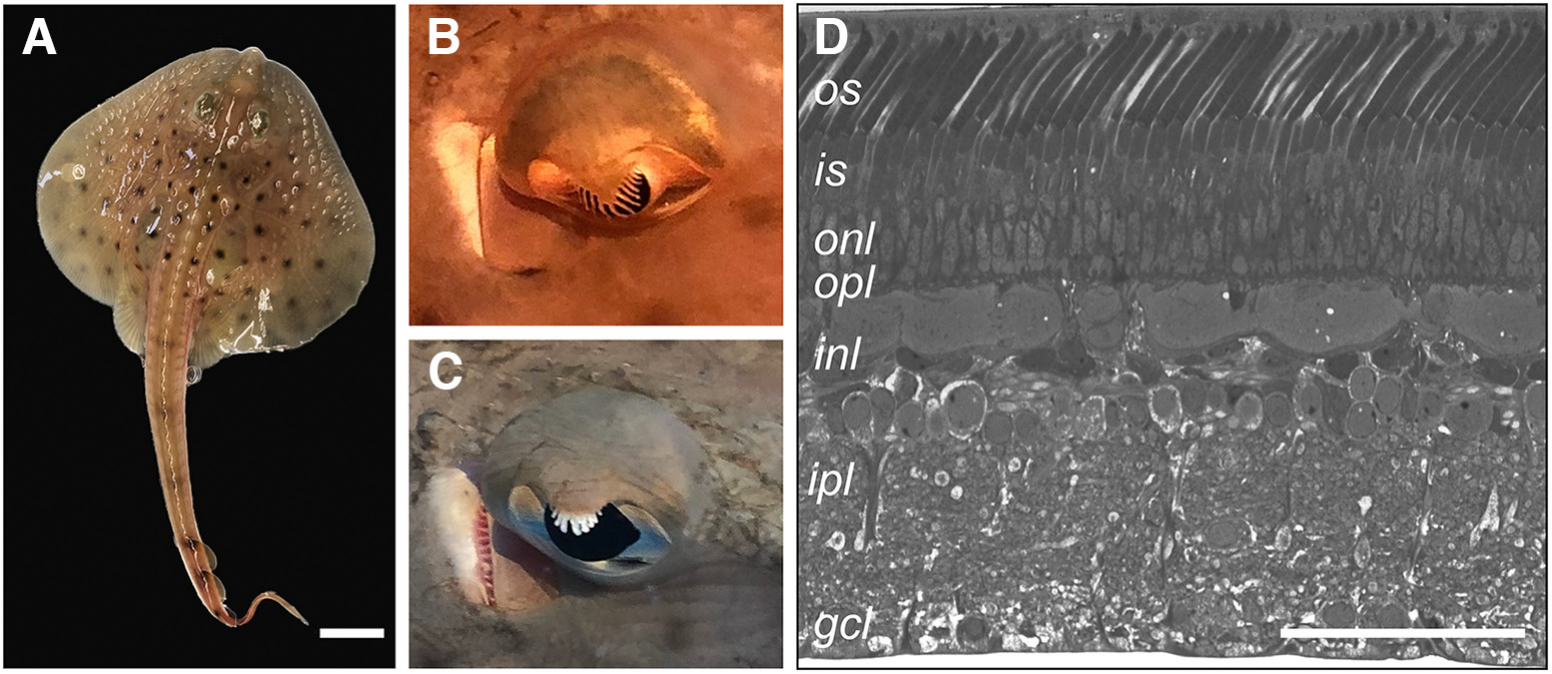
The retina of the little skate is simplex and only a single photoreceptor cell type can be distinguished by gross morphology. ***A***, A juvenile hatchling example of the little skate (*L. erinacea*). The skate is a benthic fish found off the east coast of the United States. It co-habits naturally with another species, *Leucoraja ocellata* (winter skate) and both have virtually identical appearance. Their retinas are also both simplex (scale bar = 1 cm). ***B***, The iris operculum of a light-adapted skate eye can be seen covering the majority of the cornea. ***C***, During dark-adaptation, the iris operculum completely retracts and allows for a maximum amount of light to enter the eye. The spiracle, a small, round opening, is immediately posterior to the eye (to the left of the eye in both ***B*** and ***C***). Spiracles are used for respiration and draw water into the gill chambers. The gill slits are located on the ventral side of the animal. ***D***, A histologic cross-section of the adult skate retina stained with Methylene Blue and Azure II. Only rod-like outer segments are visible in cross-section, as described previously by Dowling and Ripps (1990). The retina of the winter skate is not distinguishable from that of the little skate in any appreciable way (scale bar = 100 μm). OS, outer segments; IS, inner segments; ONL, outer nuclear layer; OPL, outer plexiform layer; INL, inner nuclear layer; IPL, inner plexiform layer; GCL, ganglion cell layer.

The elasmobranch fish *L. erinacea* (common name: little skate) is a benthic species commonly found off the east coast of the United States. A juvenile hatchling animal is ∼80–100 mm long, tip to tail, with a disk diameter of 4–5 mm (Fig. 1*A*). A very closely related species, *Leucoraja ocellata* (common name: winter skate), naturally co-habits in the same waters as *L. erinacea* and is morphologically identical. The pupil of the skate eye is covered by a structure called the operculum pupillare (Hart et al., 2006; Jinson et al., 2018). The operculum pupillare completely covers the pupil when the animal is light-adapted (Fig. 1*B*), and completely retracts to expose the whole pupil when the animal is dark-adapted (Fig. 1*C*). Both species have retinas that are termed “simplex” and contain only a single photoreceptor cell type (Fig. 1*D*). Numerous studies in both species have confirmed that their retinas are identical and simplex (Dowling and Ripps, 1990; Ripps and Dowling, 1990; Anastassov et al., 2014). Furthermore, skate photoreceptors exhibit a kind of “functional plasticity,” which allows them to seamlessly adapt to both scotopic and photopic illumination conditions (Dowling and Ripps, 1972; Carter Cornwall et al., 1989; Chappell et al., 2008). This functional trait appears to be conserved, and neurons that are downstream of photoreceptors can also adapt from scotopic to photopic conditions (and back) therefore continuing to transmit the visual message (Dowling and Ripps, 1970, 1971, 1972). For the purposes of this study, and for the reasons stated earlier, we have not differentiated between the two species.

### Serial EM imaging of the *L. erinacea* retina confirms exclusive presence of a single photoreceptor cell type

**Figure 2.**
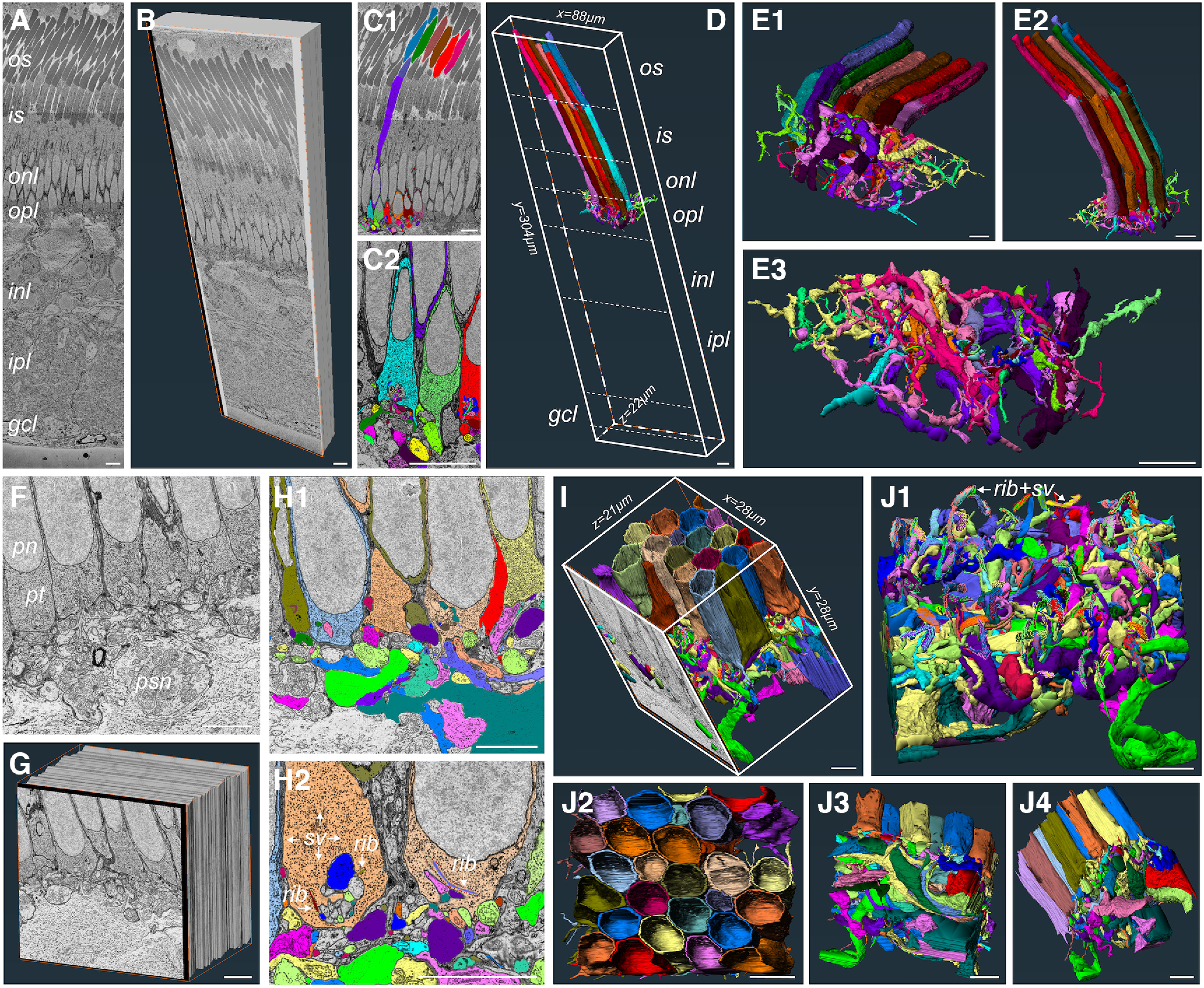
Serial block-face scanning electron microscopy (SB-3DEM) imaging and segmentation of skate retinal tissue. ***A***, Single section from full cross-section HVMS dataset. ***B***, A virtual block reconstruction of all sections within volume (scale bars = 10 μm). ***C1***, ***C2***, Examples of semi-manual segmentation of structures using Reconstruct software (scale bars = 10 μm). ***D***, 3D reconstructions of photoreceptors, where they fall within the EM volume, and the dimensions of the HVMS dataset (scale bar = 10 μm). ***E1–E3***, Side and bottom-up views of 3D reconstructions of entire photoreceptors and their associated postsynaptic dendritic processes (scale bars = 10 μm). ***F***, Single section from full cross-section P2R9 dataset; pt, photoreceptor terminal; pn, photoreceptor nucleus; psn, postsynaptic neuron; rib, synaptic ribbon; sv, synaptic vesicles (scale bar = 5 μm). ***G***, A virtual block reconstruction of all sections within volume (scale bar = 5 μm). ***H1***, ***H2***, Examples of semi-manual segmentation of structures within P2R9 volume using Reconstruct software (scale bars = 5 μm). ***I***, 3D reconstructions of photoreceptor terminals and their associated postsynaptic dendritic processes and the dimensions of the volume; one section of the raw data are also shown. ***J1***, 3D reconstructions of postsynaptic processes with photoreceptor terminals removed and photoreceptor synaptic ribbons with docked vesicles displayed (***J2–J4***) Side and top-down views of all 3D reconstructions within volume (scale bars = 5 μm).

We performed serial block-face 3D electron microscopy imaging on several retinal samples from adult skates. To our knowledge, this is the first time that this imaging approach has been applied systematically to any simplex retina. The results presented here were obtained from 2 different datasets: a full cross-section dataset (HVMS) and a high-resolution region of interest (ROI) dataset (P2R9). Figure 2*A*,*F* shows representative 2D images of each dataset. The HVMS dataset is from a full cross-section of an adult retina and a virtual stack of the volume with dimensions can be seen in Figure 2*B*,*D*. The P2R9 dataset is from an ROI in the outer plexiform layer (OPL) and a virtual stack of the volume with dimensions can be seen in Figure 2*G*,*I*. Manual and semi-manual segmentation in the outer retina from the HVMS dataset allowed us to obtain 3D reconstructions of whole photoreceptors, including inner and outer segments (IS and OS), synaptic terminals, and a significant portion of invaginating postsynaptic processes (Fig. 2*C1*,*C2*). The full volume of the HVMS dataset (with raw data excluded) and the reconstructions of 9 full photoreceptors, along with some of their connecting postsynaptic processes, can be seen in Figure 2*D*,*E1–E3*. Skate photoreceptors take up ∼50% of the cross-sectional length of the whole retina. Reconstructing nine photoreceptors and a portion of their connected postsynaptic dendritic architecture covered the full z-dimension of the HVMS dataset suggesting that a depth of 22 μm is sufficient for partial reconstruction of postsynaptic architecture, but likely insufficient for the reconstruction of full postsynaptic partners, like entire bipolar or horizontal cells, which appear to be quite a lot larger than the HVMS dataset spans in the *z*-plane. Nevertheless, we believe we have sufficient data to differentiate between different postsynaptic processes and assign them to putatively different postsynaptic photoreceptor partners. This estimation is confirmed by the significantly different morphology and spatial location of photoreceptor postsynaptic processes reconstructed from the high resolution P2R9 dataset (Fig. 2*J1*,*J3*,*J4*). Fine details of photoreceptor synaptic architecture, like ribbons, synaptic vesicles and invaginating contacts, can be distinguished readily from the high-resolution ROI dataset P2R9, a representative 2D image of which is shown in Figure 2*F*. Segmentations from different neighboring photoreceptor terminals and the postsynaptic processes invaginating into each terminal can be seen in Figure 2*H1*,*H2*. Note the marked and readily distinguishable synaptic ribbons and ribbon-docked synaptic vesicles in Figure 2*H2*,*J2*. The full R2P9 volume allowed for a full reconstruction of 20 photoreceptor terminals and partial reconstruction of another nine photoreceptor terminals (Fig. 2*I*,*J2*). Synaptic ribbon clusters could be used to readily determine the location of an individual photoreceptor terminal (Fig. 2*J1*).

### The outer segments of skate photoreceptors display separated stacked membrane morphology, which is typical for rods of duplex retinas

**Figure 3.**
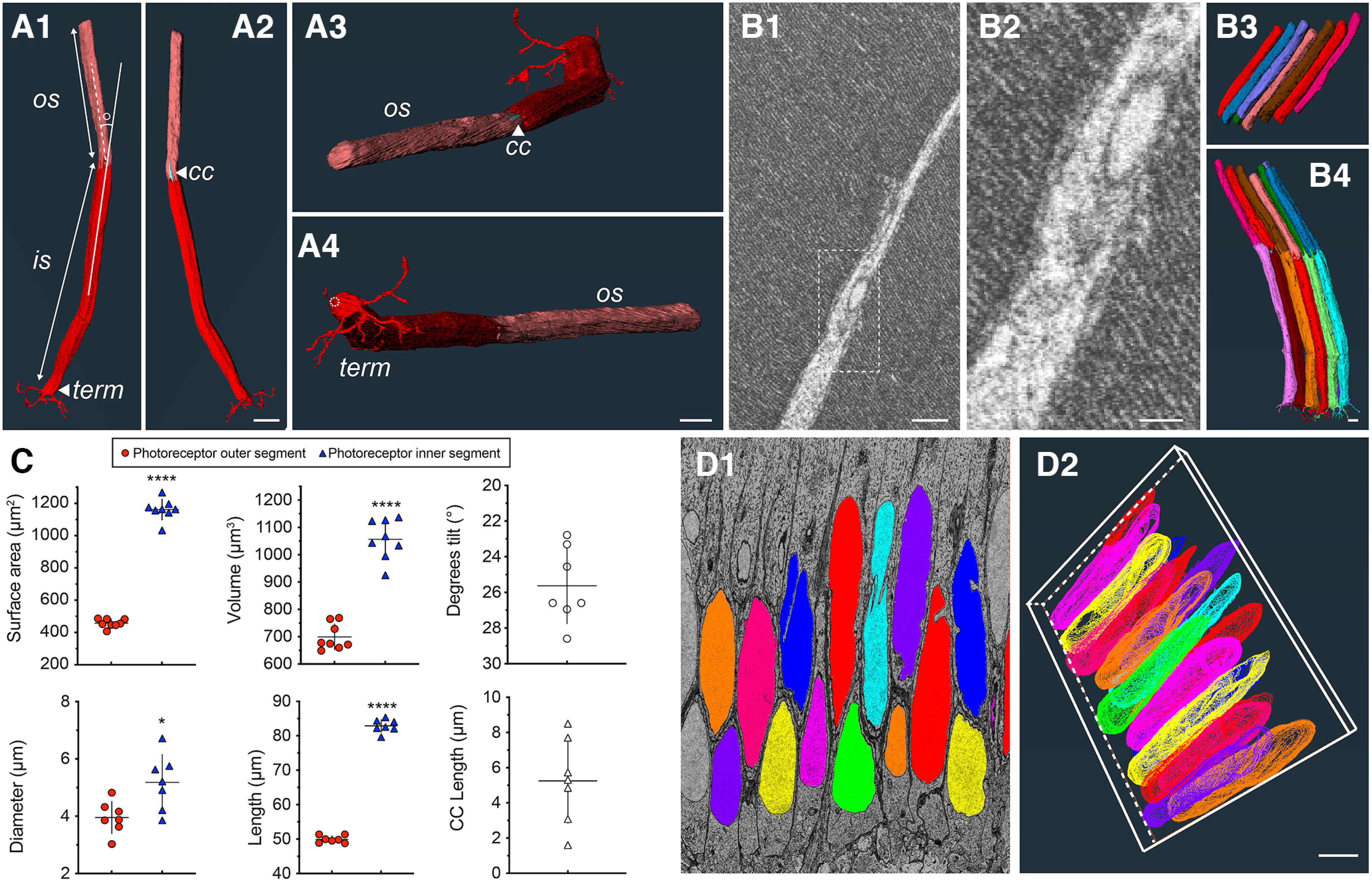
Anatomy and ultrastructure of whole skate photoreceptors. ***A1–A4***, Reconstructions of a single photoreceptor showing outer and inner segment (OS, IS), tilt angle between OS and IS, connecting cilium (CC), and synaptic terminal (term) with telodendria. The opening through which invaginating processes enter the photoreceptor terminal is off-set from the center and is indicated with a dashed circle in ***A4*** (scale bars =10 μm). ***B1***, ***B2***, Disk membrane stacking and separation of membrane discs from cell plasma membrane is readily seen in the skate photoreceptor OS, which is typical of vertebrate rods (***B1*** scale bars = 0.5 μm; ***B2*** scale bar = 0.2 μm). ***B3***, ***B4***, Relatively little stacking of outer segments or whole photoreceptor cells is seen, as opposed to murine or primate retinas (scale bars = 1 μm). ***C***, Quantifications and comparisons of photoreceptor OS and IS surface area (unpaired *t* test, two-tailed, *p* < 0.0001, *n* = 8), diameter (unpaired Mann–Whitney test, two-tailed, *p* = 0.0186, *n* = 7), volume (unpaired *t* test, two-tailed, *p* < 0.0001, *n* = 8), length (unpaired *t* test, two-tailed, *p* < 0.0001, *n* = 7), OS versus IS tilt angle, and CC lengths of fully reconstructed cells. **** in surface area, volume and length measurements designates *p* value of less than 0.0001 (*p* < 0.0001). * in diameter measurement designates a *p* value of 0.0186 (*p* = 0.0186). ***D1***, ***D2***, ONL is one to two nuclei thick, averaging ∼31.3 μm across datasets. The relatively thin ONL is similar to what is found in diurnal animals (Walls, 1942; scale bars = 5 μm).

The skate photoreceptor cell is long, which appears quite similar to the anatomy of mammalian duplex retina rods (Sterling et al., 1988; Wensel et al., 2015). Skate photoreceptors are, however, not quite as slender as mammalian rods and have a larger OS/IS diameter, which is more typical of the anatomy of rods in nonmammalian duplex retinas (Klooster and Kamermans, 2016; Witkovsky, 2000). Photoreceptors take up ∼50% of the cross-sectional length of the retina and there are clearly distinguishable outer segments (OSs), inner segments (ISs) and synaptic terminals (STs; Fig. 3*A1–A4*). Additional photoreceptor features are described in the sections that follow. High-resolution single image TEM data from Szamier and Ripps (Szamier and Ripps, 1983) has shown previously that the OS of *L. erinacea* photoreceptors have the typical ordered stacks of internal membrane disks (in all likelihood holding the proteins that take part in the rhodopsin light response cascade), which are physically separated from the photoreceptor plasma membrane. Although not of the same high resolution as conventional 2D TEM, our SB-3DEM data allowed us to confirm the finding of Szamier and Ripps of stacked internal disks separated from the photoreceptor plasma membrane (Fig. 3*B1*,*B2*). There is a slight offset in the vertical location of each OS, which can be seen in Figure 3*B3*,*B4*.

### Photoreceptor outer and inner segments vary little in diameter from each other, inner segment is consistently longer and larger, and ONL is one to two nuclei thick

We performed detailed quantitative analysis of different photoreceptor features based on the 3D reconstructions obtained. These analyses showed that inner segments were consistently longer than outer segments by ∼40% (mean of 83-μm IS vs 50-μm OS; Fig. 3*C*). Consistent with this observation, surface area (mean of 1162 μm^2^) and volume (mean of 1056 μm^3^) for IS were also significantly larger than for OS (457 and 699 μm^3^, respectively; Fig. 3*C*). Diameter of IS (measured as an average of diameters at three different points along the segments) was only moderately, although still significantly, bigger than OS diameter (Fig. 3*C*). We also measured the tilt angle between the IS and OS of each reconstructed photoreceptor and each cell showed a consistent mean tilt angle of 15.6°. The tilt angle is a measure of the angle between a continuous straight line drawn through the middle of a 3D reconstruction of the inner segment (and continuing beyond the structure), and the intersecting straight line drawn through the middle of a 3D reconstruction of the outer segment. An example of this angle measurement can be seen in Figure 3*A1*, and the tilt angle values collected are summarized in Figure 3*C*. These values fall well within the values of tilt angle measurements for skate photoreceptors within the visual streak, obtained recently by Mäthger and colleagues (Mäthger et al., 2021), which they recorded to be between 5° and 25° for the majority of photoreceptors within the horizontal streak. We take this as a good confirmation for the location our samples derive from, as the average tilt angle from our reconstructions was ∼15.6°. Tilt angle values are also in agreement with the fact that tissue for SB-3DEM imaging was taken from the tapetal area of the retina, which often overlaps with the visual streak in elasmobranchs (Hart et al., 2006). ONL thickness is only approximately one to two nuclei thick in both our histologic sections, and all of our full cross-section EM data (Fig. 3*D1*). Photoreceptor nuclei are quite elongated and appear somewhat offset from each other (Fig. 3*D2*). In the context of nocturnal and diurnal species, where nocturnal species tend to have a thick ONL spanning multiple layers of photoreceptor nuclei (Walls, 1942), and diurnal species tend to have a thin ONL spanning only several, or a single row of nuclei (Walls, 1942), the skate retina appears to be closer to the anatomy of diurnal species, despite the presence of only a single photoreceptor type. We measured an average ONL thickness of ∼31.3 μm across several of our full-cross section EM datasets.

### Photoreceptors have multiple ribbons, which are centered in clusters around a single terminal invagination

**Figure 4.**
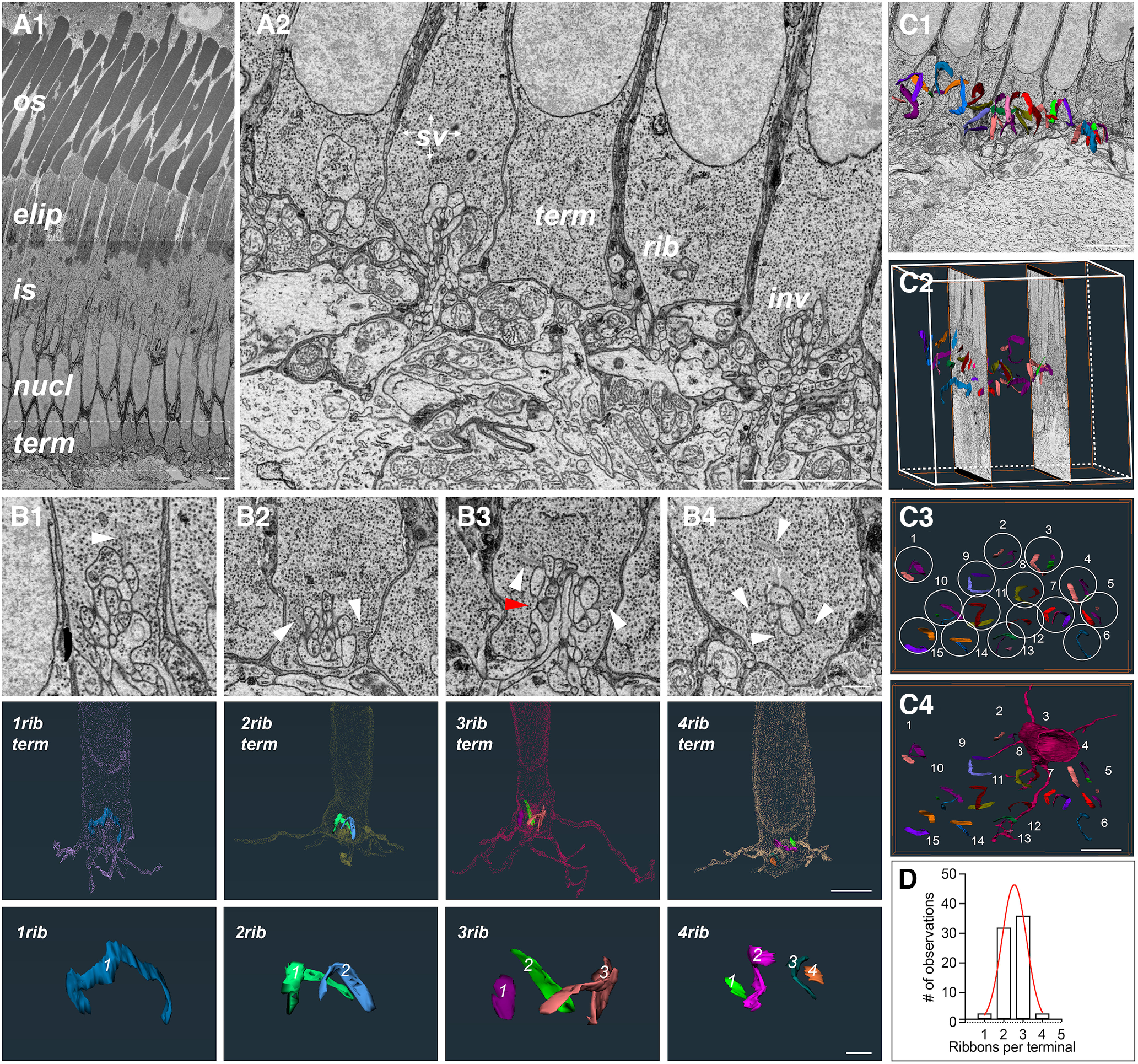
Skate photoreceptor terminals have multiple synaptic ribbons. ***A1***, A single EM image from HVMS dataset of the outer skate retina with the different areas of photoreceptors indicated; os, outer segment; elip, ellipsoid (location of mitochondrial clustering); is, inner segment; nucl, nucleus; term, synaptic terminal (scale bar = 5 μm). ***A2***, Close-up of five terminals (from left to right) from the P2R9 dataset showing some of the typical features found in skate photoreceptor terminals, as wells as some of the postsynaptic processes that invaginate into those terminals; sv, synaptic vesicles; term, terminal; rib, synaptic ribbon; inv, invagination (scale bar = 5 μm). ***B1***, An electron microscopy (EM) image with an example (white arrowhead) of a single ribbon terminal (top); 3D reconstruction of the same terminal with the one ribbon visible inside it (middle); a 3D reconstruction of the single ribbon (bottom). ***B2***, An EM image with an example (white arrowheads) of a two-ribbon terminal (top); 3D reconstruction of the same terminal with the two ribbons visible inside it (middle); 3D reconstruction of both ribbons together (note the appearance of a spherical arrangement of the ribbons over the single invagination). ***B3***, An EM image with an example (white arrowheads) of a three-ribbon terminal. The red arrowhead shows the approximate location of the third ribbon, which appears in later sections (top); 3D reconstruction of the same terminal with the three ribbons visible inside it (middle); 3D reconstruction of all three ribbons together (note again the appearance of a spherical arrangement of the ribbons over the single invagination). ***B4***, An EM image with an example (white arrowheads) of a four-ribbon terminal (top); 3D reconstruction of the same terminal with the four ribbons visible inside it (middle); 3D reconstruction of all four ribbons together (scale bars in EM images = 1 μm; scale bars in terminal reconstruction images = 5 μm; scale bars in ribbon images = 1 μm. ***C1***, ***C2***, Ribbons appear in clusters and can be seen here overlaid with individual raw EM images in 2D (***C1***) and 3D (***C2***). ***C3***, ***C4***, Photoreceptor ribbons assume a “spherical” arrangement, ribbons belonging to an individual photoreceptor terminal are circled and numbered. An example terminal belonging to the ribbons in circle 3 is shown. Note widely extending telodendria (scale bars = 5 μm. ***D***, Photoreceptor terminals have between one and four ribbons, which is quantified here in the histogram of ribbon distributions in the data to date. Red curve represents least squares Gaussian fit with best fit values: amplitude = 46.33, mean = 2.545, SD = 0.6367.

We continued our investigation of skate photoreceptor morphology by focusing on a common feature in the terminals of primary sensory neurons: synaptic ribbons. These organelles serve as organizing centers that tether synaptic vesicles at the active zones of photoreceptor and bipolar cell terminals in the vertebrate retina (Sterling and Matthews, 2005; LoGiudice and Matthews, 2009). Synaptic ribbons in the vertebrate retina have been studied extensively (Jackman et al., 2009; Wan et al., 2010; Grassmeyer and Thoreson, 2017), and in mammals, the synaptic terminal of rod spherules tends to contain one ribbon centered around a single terminal invagination (Rao-Mirotznik et al., 1995, 1998; Zimov and Yazulla, 2004). In some fishes and amphibians, however, rods can contain more than one ribbon (Kock and Stell, 1985; Witkovsky, 2000). Mammalian cone pedicles tend to contain multiple ribbons and invaginations, where each ribbon is centered over its dedicated invagination, but all are contained in the same terminal (Haverkamp et al., 2000; Strettoi et al., 2010). Teleost and amphibian cone pedicles vary and can sometimes have a single large invagination surrounded by multiple ribbons (Klaassen et al., 2016). Surprisingly, skate photoreceptor terminals exhibit a morphology that is in between that of a typical vertebrate rod and cone. That is, there is a single invagination with multiple ribbons centered around it (Fig. 4*A1*,*A2*). Furthermore, the number of ribbons clustered over the invagination is not constant and we repeatedly encountered terminals with either one, two, three, or four ribbons (Fig. 4*B1–B4*). Ribbon clusters assume a spherical arrangement over the invaginating postsynaptic processes, covering it like an umbrella (Fig. 4*C1*,*C2*). We examined 74 rod terminals across the HVMS and P2R9 datasets (as well as a third partial ROI dataset not shown here) and found that ribbon distribution is heavily skewed toward terminals with two (*n* = 32) or three ribbons (*n* = 36), with one or four ribbons (*n* = 3, for both) at the tail end of the distribution and a relatively rare occurrence (Fig. 4*D*). The spatial arrangement of terminals with different number of ribbons did not show any appreciable pattern (Fig. 4*C3*,*C4*) and a correlation between the number of ribbons and the number of invaginating processes is largely lacking.

### Photoreceptor terminals have multiple telodendria that extend to form a meshwork

**Figure 5.**
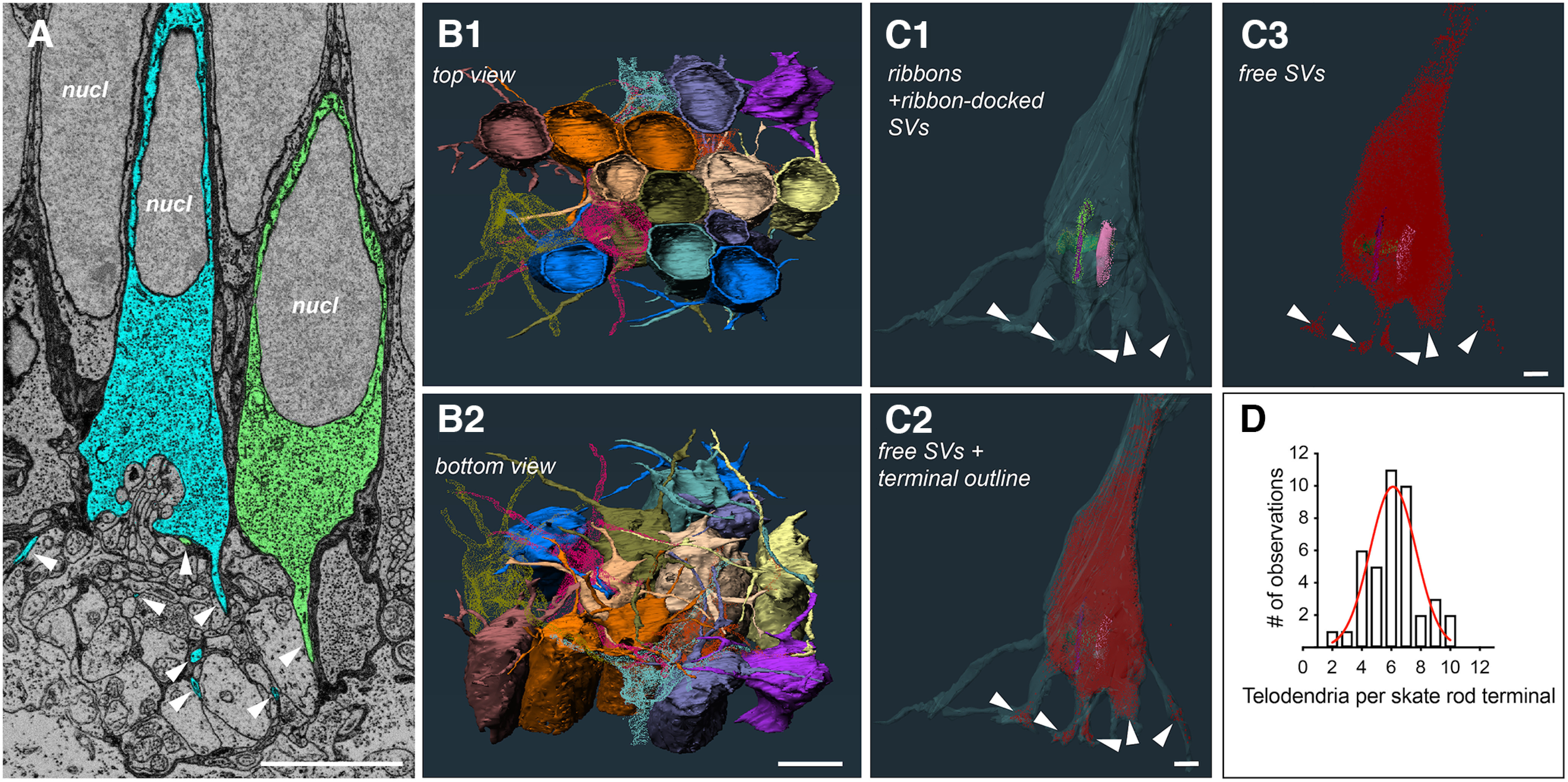
Skate photoreceptor terminals have a hybrid terminal morphology, with multiple extending telodendria, similar to mammalian cones. ***A***, Single section from HVMS volume showing segmentation of two neighboring photoreceptor terminals and their extending telodendria in cyan and green (white arrowheads; scale bar = 5 μm); nucl, nucleus. ***B1***, ***B2***, Top and bottom view of partial photoreceptors with reconstructed terminals and the extending telodendria of each. Several of the terminals and their telodendria have been made transparent and not all terminals are shown for clarity (scale bar = 5 μm). ***C1***, A transparent 3D reconstruction of a single terminal with ribbons and ribbon-docked vesicles visible, some the endings of telodendria containing free synaptic vesicles are indicated by white arrowheads. ***C2***, All free vesicles within the same terminal. ***C3***, Vesicles are often observed at the ends of telodendria (white arrowheads), suggesting synapses onto other photoreceptors or second-order cells (scale bar = 1 μm); SV, synaptic vesicle. ***D***, Distribution of telodendria numbers from all reconstructed terminals across both datasets. Red curve represents least squares Gaussian fit with best fit values: amplitude = 9.949, mean = 6.120, SD = 1.561.

**Figure 6.**
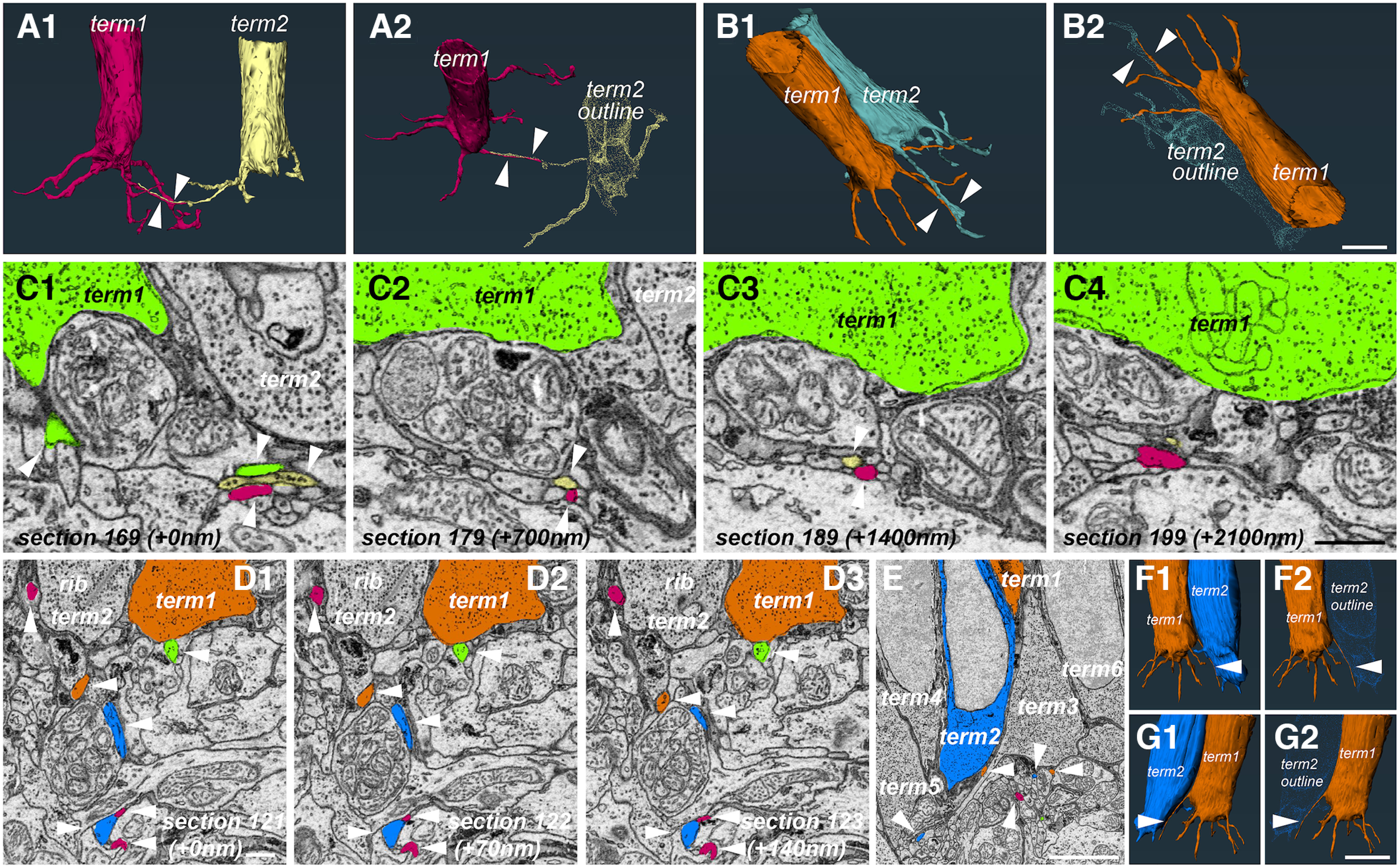
Telodendria association between nonadjacent and adjacent photoreceptors. ***A1***, ***A2***, 3D reconstructions of 2 terminals separated by ∼20 μm from the P2R9 dataset showing their respective telodendria extending toward each other and running adjacent to each other (white arrowheads) for ∼10 μm indicating a possible association via a nonchemical synapse connection. ***B1***, ***B2***, 3D reconstructions of two immediately adjacent photoreceptor terminals from the same dataset. Note the close proximity of both telodendria to each other (white arrowheads) and the lack of any other telodendria with such close association in these two terminals (scale bar = ∼5 μm). ***C1–C4***, EM images showing a progression of 700-nm steps through the data (equal to 10 sections) and a demonstration of the close proximity (white arrows) of the telodendria from the photoreceptors in ***A1***, ***A2***, A telodendrion from the green terminal can also be seen in ***C1*** running in parallel for a short distance. Possible synaptic vesicles in the yellow telodendrion can be seen in ***C1*** as well. Thirty sections later (i.e., ∼ 2100 nm), the red and yellow telodendrion separate (scale bar = 1 μm). ***D1–D3***, A possible basal contact between the telodendrion of one terminal (green + white arrowhead) and the terminal of a nonadjacent photoreceptor (dark orange). Other white arrowheads show telodendria from neighboring photoreceptors crisscrossing the IPL; rib, synaptic ribbon; term, synaptic terminal (scale bar = 1 μm). ***E***, A telodendrion from a more distally positioned photoreceptor (term1, dark orange) extending past an adjacent photoreceptor (term2, blue) but remaining in close proximity to the soma suggesting possible contact. White arrowheads point to telodendria from the blue, dark orange and burgundy terminals (scale bar = 5 μm). ***F1***, ***F2***, ***G1***, ***G2***, 3D reconstructions of the blue and dark orange terminals from different angles showing the proximity between the blue terminal and the dark orange terminal telodendrion (scale bar = 5 μm).

The terminals of mammalian rod and cone photoreceptors differ from each other in a number of morphologic features, one of them being the telodendria extending from the cone pedicles (Behrens et al., 2016). Usually, mammalian cone pedicles have multiple long extensions (i.e., telodendria), which may connect them to neighboring cones, or other postsynaptic cells within the local circuitry (Ishibashi et al., 2022). Mammalian rod spherules tend to lack telodendria and connect to each other, or other cones, via gap junctions (Sterling et al., 1986; Behrens et al., 2016). However, nonmammalian rods and cones are more diverse, with rod and cone telodendria varying in numbers and lengths for each photoreceptor type, based on species (Ramon y Cajal, 1995; Asteriti et al., 2015; Noel and Allison, 2018). All photoreceptor terminals that we examined and reconstructed for telodendria across our datasets (*n* = 41), had a number of long and extending telodendria, as can be seen from the example segmentation from the raw data in Figure 5*A*. 3D reconstructions of telodendria also show that they form an intricate meshwork of processes between photoreceptors (Fig. 5*B1*,*B2*). We analyzed 41 full photoreceptor terminals and the distribution of the number of telodendria per photoreceptor terminal is shown in Figure 5*D*. We encountered terminals with six or seven telodendria most often, while the length of each process, within and between terminals, varied considerably and was not clearly correlated with how many telodendria per terminal were present (Fig. 6). Putative synaptic vesicles were identified in multiple locations along the length of different telodendria, suggesting that there are multiple synaptic contacts that telodendria make to neighboring processes (Fig. 5*C1–C3*). These processes appeared to be either other telodendria, or the putative dendrites of postsynaptic cells.

The prevalence of synaptic vesicles along telodendria seems unusual, as they are assumed to mostly interconnect photoreceptors via gap junctions, not chemical synapses (J.J. O’Brien et al., 2012). Unfortunately, we seem to lack the resolution in our current skate retina datasets to be able to confidently identify gap junctions between individual photoreceptor terminals, or between the telodendria of different photoreceptors.

### Telodendria of different photoreceptors often run parallel to each other and come close to adjacent photoreceptor terminals to form putative basal contacts

Aside from the intricate, and likely connected, meshwork that photoreceptor telodendria formed, we occasionally encountered another unusual characteristic, namely, a telodendrion from one photoreceptor was in close apposition to the telodendrion of another photoreceptor, often over significant distances (Fig. 6). This could be observed in photoreceptors that are immediately next to each other (Fig. 6*B1*,*B2*), or photoreceptors that were separated by 20 or more micrometers from each other (Fig. 6*A1*,*A2*,*C1–C4*). Occasionally, we could also identify putative synaptic vesicles in some of these processes (Fig. 6*C1*). As mentioned in the previous sections, our current data does not have enough resolution to definitively identify gap junctions, but we suggest that the close proximity of telodendria from adjacent and nonadjacent photoreceptors is not random, but rather indicative of contacts. We also observed instances of close association resembling basal contact between the extending telodendrion of one photoreceptor and the terminal base of a nonadjacent photoreceptor, with a flattening of membranes between the telodendrion and the terminal, strongly suggesting association (Fig. 6*D1–D3*). Telodendria from more distally located photoreceptors also appear to be in close apposition directly to the terminal membrane of an adjacent photoreceptor for extended distances, suggesting contact from the adjacent photoreceptor (Fig. 6*E*,*F1*,*F2*,*G1*,*G2*). It is also possible that the telodendria of some photoreceptors invaginate into the terminals of adjacent photoreceptors, as has been observed in zebrafish (Fadool, 2003). Although we have had several preliminary observations (data not shown) that suggest this might be happening for skate photoreceptors as well, we have not been able to confidently confirm that this is the case.

### Multiple postsynaptic processes invaginate into a single skate photoreceptor terminal forming structures that are unlike the typical tetrad observed in the terminals of rods from duplex retinas

**Figure 7.**
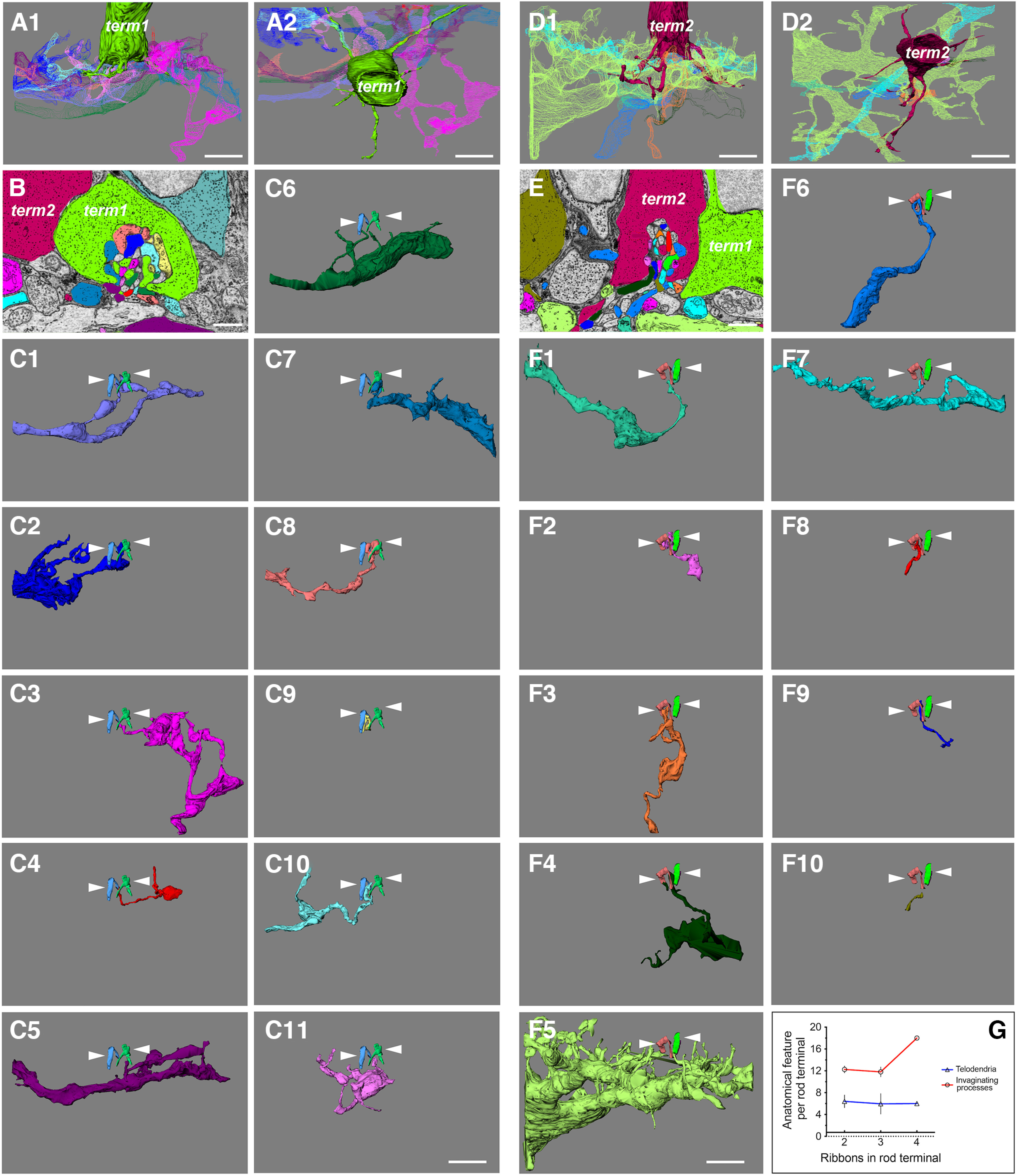
Multiple postsynaptic processes invaginate into a single photoreceptor terminal. ***A1***, ***A2***, Side and top-down view of a partial photoreceptor terminal from the P2R9 dataset along with all of its reconstructed invaginating postsynaptic contacts (scale bar = 5 μm). term, synaptic terminal; EM, electron microscopy. ***B***, EM image and segmentation example of the same terminal (term1, green) and its invaginating processes (scale bar = 1 μm). ***C1–C11***, 3D reconstructions of each invaginating process with the photoreceptor terminal removed for clarity. White arrowheads point to the two synaptic ribbons of the green terminal. At least 11 distinct processes appear to invaginate into this terminal (scale bar = 5 μm). ***D1***, ***D2***, Side and top-down view of a partial photoreceptor terminal from the P2R9 dataset along with all of its reconstructed invaginating postsynaptic contacts (scale bar = 5 μm). ***E***, EM image and segmentation example of the same terminal (term2, burgundy), and its invaginating processes scale bar = 1 μm). ***F1–F10***, 3D reconstructions of each invaginating process with the photoreceptor terminal removed for clarity. White arrowheads point to the three synaptic ribbons of the burgundy terminal. At least 10 distinct processes appear to invaginate into this terminal. ***B*** and ***E*** show that these two terminals are adjacent to each other but do not appear to share any of the reconstructed processes, with the possible exception of process ***F5*** of term2 (scale bar = 5 μm). ***G***, A graph of the number of postsynaptic processes we have segmented from different terminals. There is no appreciable correlation between the number of ribbons and the number of invaginating processes for any given terminal (data for terminals with four ribbons is insufficient for any conclusions (*n* = 1). Photoreceptor telodendria are also not significantly different between terminals with different number of ribbons and different number of invaginating processes.

Processes from vertebrate postsynaptic retinal neurons, namely bipolar cells and horizontal cells, tend to invaginate into the synaptic terminal of their target photoreceptor, be it rod or cone (for some of many examples see Stell et al., 1977; Haverkamp et al., 2000; Witkovsky, 2000; Tsukamoto et al., 2001; Tsukamoto and Omi, 2014). This anatomy tends to be somewhat stereotypical and in mammalian rod terminals, often called spherules, four invaginating processes come from two horizontal and two bipolar cells, forming a so called “triad” or “tetrad” synapse (Kolb, 1970, 1977, 1994; Kolb and Jones, 1984). These processes terminate at different invaginating depths under a single ribbon with the horizontal cell dendrites almost invariably closer to the ribbon and the bipolar cell dendrites more distant. However, variations and exceptions on this theme in mammalian retinas are beginning to be described (Tsukamoto and Omi, 2022). On the other hand, mammalian cone terminals, often called pedicles, display a seemingly quite different anatomy, with multiple ribbons and multiple invaginating processes (Strettoi et al., 2010). Upon closer examination, it becomes apparent that each synaptic ribbon bears a striking resemblance to a single rod spherule, i.e., there are two bipolar cell processes and two horizontal cell processes terminating under each individual ribbon. The whole mammalian cone pedicle, then, resembles a number of spherules brought together in a single terminal. In nonmammalian retinas, the individual anatomy of rod and cone terminals is similar, but there are some differences, depending on the species. For example, multiple ribbons seem to be present in rod terminals of amphibian rods (Witkovsky, 2000) and cone pedicles have a single large invagination (Klaassen et al., 2016). Surprisingly, our very close inspection of the skate photoreceptor terminal revealed a different organization. Some of it we have already described in the previous sections (see section describing ribbons). What we noticed here, is the presence not only of multiple ribbons in each terminal, but also of multiple invaginating processes (Fig. 7*B*,*E*). Each photoreceptor terminal we examined did not have four invaginating processes, as would be expected from other work examining vertebrate rod terminals, but a much higher number, all clearly invaginating into that terminal. The partially reconstructed invaginating postsynaptic process of two neighboring photoreceptor terminals from the P2R9 volume are shown in Figure 7*A1*,*A2*,*D1*,*D2*. The individual postsynaptic processes for each terminal are shown individually in Figure 7*C1–C11*,*F1–F10*.

Our quantifications show that there are at least 10, but as many as 18, distinct invaginating processes that we could reasonably assign to reconstructed postsynaptic partners in different photoreceptor terminals. This is likely a conservative estimate, since we sometimes had to omit processes we thought were not sufficiently traced to establish them as anatomically distinct. Interestingly, there does not seem to be any correlation between the number of telodendria per photoreceptor terminal, and the number of invaginating processes (Fig. 7*G*). Because of the rare occurrence of terminals with four ribbons, we only have one such terminal with reconstructed invaginating processes, where there appear to be 18. This is insufficient data for us to make any definitive conclusions about a correlation between the number of ribbons and the number of invaginating processes for terminals with four ribbons and further investigation is necessary. However, terminals with two and three ribbons appear to have very similar number of invaginating processes (*n* = 4, mean = 12.25 for two ribbons; *n* = 5, mean = 11.80 for three ribbons; Fig. 7*G*). We currently do not have reliable data for terminals with one ribbon, as they are also rare and additional imaging is needed to capture all invaginating processes in such terminals.

## Discussion

In this study, we report a number of ultrastructural hallmarks of the functionally plastic photoreceptor of the skate retina. Using serial EM imaging, we show that skate photoreceptors display typical rod characteristics in their outer segments, but somewhat hybrid rod-cone characteristics in their inner segments and synaptic terminals. Thus, the skate photoreceptor almost appears to be separated into two distinct anatomic domains. Namely, outer segment architecture displays stacked membrane discs physically separated from the plasma membrane, as in a typical rod, and likely reflective of the physiology and ability of skate photoreceptors to function with great sensitivity at scotopic light levels (Carter Cornwall et al., 1989). On the other hand, skate photoreceptors’ synaptic architecture appears to borrow elements of cone pedicle design, perhaps reflective of their ability to recover functionality under photopic light conditions (Dowling and Ripps, 1970).

### The little skate as a novel model in the study of the comparative neuroscience of vision

The physiology and molecular landscape of the duplex (i.e., mixed rod-cone) vertebrate retina have been worked out in great detail in the past ∼50 years, especially for mammals (Nilsson, 2009; Baden and Euler, 2013; Demb and Singer, 2015; Lamb, 2019). Transgenic approaches in animal models have also contributed greatly to our study of different elements of rod and cone circuitry, together, or in isolation (Soucy et al., 1998; Mears et al., 2001). However, a large number of studies of the visual system have been performed on a fairly limited number of model organisms, like mouse, rat, rabbit, zebrafish, or salamander. This is likely because technical approaches have already been well established for these organisms, but it is worth noting that the almost exclusive study of the visual system in such model organisms has perhaps given us too narrow a focus and may have introduced bias in our understanding of broad and comparative principles of retinal design. The simplex, single photoreceptor cell type, skate retina provides us with an important new comparative model and an exciting avenue to study vertebrate photoreceptor circuitry within the context of a system well-adapted to function in its ecological niche. Furthermore, the ability of this simplex retina to perform work under both scotopic and photopic ranges of illumination allows us a unique opportunity to examine how complete our understanding of retinal design and physiology is. Indeed, only in the last several years, a long-held assumption that rods saturate and are mostly inactive under photopic conditions in a duplex retina has been challenged (Tikidji-Hamburyan et al., 2017; Frederiksen et al., 2021). Thus, such novel comparative models should not be underestimated, as they have the potential to add exciting new avenues in vision restoration efforts and to aid our overall understanding of the vertebrate visual system.

### Similarities and differences between rods and cones from duplex retinas and photoreceptors from the simplex retina of skate

#### Outer segments, inner segments, and synaptic terminals

Our detailed examination of the outer segments of skate photoreceptors using a SB-3DEM approach has confirmed the typical architecture of stacked membrane disks separated from the plasma membrane, as observed previously by Szamier and Ripps (Szamier and Ripps, 1983), and typically expected of vertebrate rods from mammalian and nonmammalian retinas, alike (Rodieck, 1998). This architecture is different from vertebrate cones, which have an outer segment plasma membrane and stacked membrane disks that are continuous with each other (Mustafi et al., 2009). These outer segment structural hallmarks were long thought to be important mediators of function in both rods and cones, but an elegant recent study in lamprey retina by Morshedian and Fain (Morshedian and Fain, 2015) definitively showed that the single-photon sensitivity of rods is not intimately connected to the separation of outer segment disk membranes and the rod plasma membrane, since lamprey rods have cone-like outer segments. Skate photoreceptors appear to have sensitivity approaching single photon detection (Carter Cornwall et al., 1989) and, despite their functional plasticity and ability to light-adapt to photopic levels of illumination, they display a stereotypical outer segment morphology, as we have confirmed here. Yet, strikingly, they still retain an ability to speed up their kinetics, lower their sensitivity, and expand their functional capabilities to cone levels of illumination. We propose that some of that remarkable capability has structural underpinnings manifested in the inner segment and synaptic terminals of skate photoreceptors. We have elaborated on this hypothesis in the sections that follow.

It is worth noting that retinal samples for this study were dissected under ambient illumination and thus we should consider the photoreceptors light-adapted (see Materials and Methods). Teleost fish and amphibians undergo diurnal and circadian retinomotor movements, which generally manifest themselves in changes of the myoid portion of the inner segment of each photoreceptor type (Welsh and Osborn, 1937; Besharse et al., 1977; Menger et al., 2005). Specifically, light-adapted rod inner segments elongate, thereby bringing outer segments closer to the retinal pigment epithelium (RPE), while cone inner segments retract from the RPE in the light-adapted state; the situation is reversed for each photoreceptor type during dark-adaptation. Although to the best of our knowledge similar detailed studies of retinomotor movements in skate have not been performed, a similar phenomenon has nevertheless been described for skate photoreceptors, showing a retraction of outer segments from the RPE after light offset (Szamier and Ripps, 1983). Similarly, a depletion of synaptic vesicles in the photoreceptor terminal and at the synaptic ribbon, as well as a distortion of the synaptic terminal membrane, have all been described previously for skate photoreceptors under high K^+^ perfusion conditions, i.e., maximal membrane depolarization (Ripps and Chappell, 1991). Thus, we should consider that our study likely reports skate photoreceptor inner, and probably outer, segments in their most extended state. Considering the above-cited studies, skate photoreceptor synaptic vesicles and synaptic ribbons will likely be also more numerous and longer, respectively, under light-adapted conditions. However, further detailed ultrastructural studies of these cells under these conditions are necessary to confirm these ideas.

In the results presented here, we show that skate photoreceptor terminals have a varying number of ribbons (between one and four, and possibly more) and that there is a skewed distribution toward two to three ribbons/terminal, among the photoreceptor terminals we examined. We propose that photoreceptors with different numbers of ribbons may be functionally distinct. That is, photoreceptor synaptic terminals with one or two ribbons may be more frequently used under scotopic conditions (i.e., rod-like function), while synaptic terminals with three or four ribbons may be used more heavily under photopic conditions (i.e., cone-like function). Our structural data (Fig. 4) suggests that the distribution of terminals with different number of ribbons is in a mixed mosaic, rather than a spatial clustering of terminals with specific numbers of ribbons. Furthermore, we think it is possible that ribbon structure and synaptic vesicle density may change differentially during light and dark adaptation in photoreceptors with different number of ribbons, as has been described for rods and cones in the goldfish retina (Yazulla and Studholme, 1997).

Similarly, additional preliminary data from our laboratory not presented here points to a nonselective connection of putative bipolar and horizontal cells to photoreceptors with different number of ribbons. In fact, it appears that neurons postsynaptic to photoreceptors might make connections to photoreceptors based on what is available in their dendritic field, rather than selectivity based on a specific photoreceptor attribute, at least that we can determine so far from preliminary data. As mentioned before, previous literature indicates that other vertebrate species, like teleost fish (Stell et al., 1977; Oel et al., 2020) and mammals (Carter-Dawson and Lavail, 1979; Zampighi et al., 2011), have a single synaptic ribbon per rod, although exceptions for mammals have been described (Migdale et al., 2003). Amphibians, on the other hand, tend to have multiple ribbons in their rods (Townes-Anderson et al., 1985; Usukura and Yamada, 1987; Witkovsky, 2000). However, systematic studies of rod synaptic ribbons in different vertebrate retinas are largely absent from the literature and a description of these organelles is often in a functional context, or in the context of describing structural motifs of connectivity. To our knowledge, a systematic quantification of ribbon distribution across photoreceptors in a simplex retina, as we have performed here, is completely lacking.

#### Telodendria with synaptic vesicles and gap junctions

The multiple telodendria extending from each skate photoreceptor terminal were another intriguing finding from our study. Telodendria appear to be often associated with mammalian and nonmammalian cones (Behrens et al., 2016; Noel and Allison, 2018)^,^ where they are largely believed to be sites of cone-to-cone gap-junctional contacts (J.J. O’Brien et al., 2012). Mammalian rods seem to lack telodendria (Asteriti et al., 2015), but at least some nonmammalian rods have extensive telodendria networks (Fadool, 2003) . Our results also strongly suggest the presence of conventional chemical synapses along skate photoreceptor telodendria (Fig. 5). This type of architecture might be in place of, or in addition to, gap junctional contacts along these same telodendria. At present, we do not have sufficient resolution in our data to exclude or confirm the presence of gap junctions along these processes, or between the terminal endings of adjacent photoreceptors. Nevertheless, we suggest that the role of photoreceptor telodendria in skate retina is to indeed mediate some form of receptor coupling and therefore possibly improve sensitivity (Fain et al., 1976), either through chemical or electrical synapses, or both.

#### Invaginating contacts

Yet another surprising finding in this study has been the number of individual postsynaptic processes we were able to identify in the single invagination of skate photoreceptors (Fig. 7). The high number of invaginating processes (mean of 12.85 processes per photoreceptor terminal) is not typical of vertebrate rods, yet the single invagination into the terminal is. For example, mammalian rods typically have a single invagination with four dendritic processes (evenly split between horizontal and bipolar cells) terminating proximally and distally to the synaptic ribbon, respectively (Rao-Mirotznik et al., 1995). On the other hand, mammalian cone pedicles tend to have multiple ribbons (exact number depends on the cone type) and multiple invaginations, each with approximately four invaginating processes (Haverkamp et al., 2000). This arrangement is somewhat less organized in nonmammals, but generally, there are fewer invaginating process in rods (Kock and Stell, 1985; Witkovsky, 2000) and more in cones (Dowling, 1970; Ishida et al., 1980; Kamermans et al., 2001). To our knowledge, the ultrastructural characteristics of synaptic terminal arrangement of photoreceptors in a simplex retina has never been described.

#### Structure of vertebrate rod and cone photoreceptors

Ciliary photoreceptor cells in the vertebrate retina have for a very long time been separated into two distinct classes: rods and cones (Walls, 1934). This separation was initially entirely based on strikingly different structural characteristics, like outer segment morphology for example, but gradually evolved to include a variety of physiological and molecular characteristics (Ingram et al., 2016). However, increasing number of studies in recent years have demonstrated that structural characteristics alone appear to be woefully insufficient to assign a rod or cone identity to a particular photoreceptor. For example, lamprey rods achieve single-photon sensitivity with cone-like outer segments (Morshedian and Fain, 2015), while some of the photoreceptors in the all-cone retina of garter snakes exhibit what would be considered a rod-like outer segment with stacked membranes separated from the plasma membrane (Schott et al., 2016). As we have confirmed here, skate photoreceptors have a typical rod-like outer segment, and yet, as others have shown (Carter Cornwall et al., 1989), they can effortlessly crossover into the cone functional domain. It would be, therefore, quite informative to determine whether changes in structure accompany this physiological adaptation in skate photoreceptors.

#### Considerations of skate photoreceptors and photoreceptor densities of duplex retinas

Using histologic approaches in adult animals, photoreceptor density in the skate retina was estimated by Mäthger and colleagues (Mäthger et al., 2021) to ∼7.7 × 10^4^ to ∼9.4 × 10^4^ cells/mm^2^ in the area of the visual streak (our samples are also from that approximate area). We should note that the eyes of teleost fishes continue to grow throughout life and continue to add new photoreceptors in the retinal periphery (Kunz, 2007). We assume the same is true for skate eyes, since we know that they change size as the animal grows (Anastassov IA, unpublished observations). We do not think we can obtain reliable photoreceptor densities from our data, as our EM samples do not cover a large enough area, but we can compare the photoreceptor densities from Mäthger and colleagues to average photoreceptor densities reported by others in commonly used species. Rough approximations of photoreceptor densities in zebrafish, mouse, salamander, and *Xenopus* from the literature are as follows: (1) zebrafish ∼6.0 × 10^4^ and ∼2.1 × 10^4^ cells/mm^2^ for rods and cones, respectively (Huckenpahler et al., 2018; Van houcke et al., 2019); (2) mouse ∼3.3 × 10^5^ to 5.1 × 10^5^ and ∼8.7 × 10^4^ to 1.6 × 10^5^ cells/mm^2^ for rods and cones, respectively (Jeon et al., 1998); (3) salamander ∼4.4 × 10^3^ and ∼3.5 × 10^3^ cells/mm^2^ for rods and cones, respectively (Zhang and Wu, 2009); (4) *Xenopus* ∼7.3 × 10^3^ and ∼6.4 × 10^3^ cells/mm^2^ for rods and cones, respectively (Gábriel and Wilhelm, 2001). Note, this is only an approximation from the literature and is taken across retinal eccentricities and cone spectral sensitivities, where described. It appears, albeit somewhat surprisingly, that skate photoreceptor density in the visual streak is an order of magnitude higher than what is found for both rods and cones in amphibian retinas. This holds even for photoreceptor densities away from the visual streak, based on values reported by Mäthger and colleagues (Mäthger et al., 2021). Skate photoreceptors are marginally denser in the visual streak than the total populations of zebrafish rods and cones, but that difference starts to disappear when one moves away from the visual streak and toward the periphery. Unsurprisingly, mouse rods have a density that is an order of magnitude higher, while mouse cones are of similar, or higher density than skate photoreceptors. Thus, skate photoreceptor densities appear to be higher than rod and cone densities in amphibians and teleost fishes, but generally lower than rod, and possibly cone, densities in mice. This difference could be a function of the difference in photoreceptor and eye sizes between species, and makes it difficult to determine whether the density is in some way relevant to the previously observed dual physiological function in skate photoreceptors. A careful examination of the photoreceptor mosaic and the possible electrical/chemical coupling between skate photoreceptors, as we have shown in this study, could help us understand any network contribution to said dual physiological function in skate retina.

#### Considerations of skate photoreceptor structure with relation to phototransduction adaptation

The physiology of light-adaptation in skate photoreceptor outer segments was beautifully demonstrated in single-cell suction recordings by Cornwall and colleagues (Carter Cornwall et al., 1989). We have also confirmed in the present study the outer segment structure findings by Szamier and Ripps (Szamier and Ripps, 1983; i.e., OS membrane discs separated from the plasma membrane; see Fig. 3*B1*,*B2*). It is also worth considering that molecular mechanisms of light-adaptation could involve key residue changes in skate rhodopsin, as compared with other vertebrate opsins and described by O’Brien and colleagues (J. O’Brien et al., 1997). In their study, O’Brien and colleagues cloned opsin cDNA from the skate retina and showed that there are residue changes in skate opsin at positions known to interact with phototransduction cascade proteins. For example, a residue change in skate opsin (Gln150Ser), which is considered the site for transducin interaction, might affect transducin activation levels, or even transducin translocation to the inner segment. Importantly, transducin translocation to the IS during light-adaptation has not been demonstrated for skate photoreceptors thus far. However, if such transducin translocation does take place, as has been shown for the physiology of mouse rods (Lobanova et al., 2007; Frederiksen et al., 2021), it perhaps leads to changes in phosphodiesterase 6 (PDE6) activation and changes in outer segment cGMP-gated current. In our study, we show that skate photoreceptors have inner segments that are ∼40% longer than outer segments (Fig. 3), which, along with the Gln150Ser substitution in skate rhodopsin, could have an effect on any hypothetical transducin translocation and therefore have an effect on the point at which skate photoreceptors switch from high sensitivity to low sensitivity.

### Skate photoreceptors and downstream circuitry exhibit unusual architecture previously undescribed in a single photoreceptor cell type

In this study we show that skate photoreceptors have a number of unusual structural characteristics housed in the same cell, including multiple synaptic ribbons centered over a single photoreceptor synaptic invagination, long extending telodendria that form intricate networks, and multiple invaginating contacts into each photoreceptor terminal. Interestingly, studies by several groups examining the *rd7* mutant mouse line, a model for the human disease enhanced S-cone syndrome (ESCS), demonstrate that knocking out the rod-specific nuclear receptor Nr2e3, which works in concert with the transcription factor Nrl, suppresses expression of cone-specific genes in developing rods and results in a “hybrid” photoreceptor type with both rod and cone morphologic and molecular characteristics (Chen et al., 2005; Corbo and Cepko, 2005; Peng et al., 2005). The evolutionarily conserved sequence for Nr2e3, especially in zebrafish, makes it an interesting target to search for in skate retina. Surprisingly, however, we have not been able to find Nrl expression in our recently obtained skate retina transcriptome data (Anastassov IA, unpublished observations).

While earlier classical studies have examined the physiology of the skate retina in considerable detail (Dowling and Ripps, 1970, 1971, 1972, 1977; Kline et al., 1985; Carter Cornwall et al., 1989; Malchow et al., 1990), with this study we aim to build toward a similar comprehensive description of the underlying retinal ultrastructure. Our observations should offer a valuable contribution to our understanding of the anatomic organization of simplex retinas at large, especially since serial ultrastructural data from such retinas are largely lacking. Future studies exploring the structure and characteristics of the inner skate retina using SB-3DEM methodology, as well as serial EM examinations of the photoreceptors and inner retina in other understudied vertebrate species, are needed. Findings from such endeavors should help us build a comprehensive understanding of what, if any, are the defining structural features of photoreceptors and other cell types in vertebrate retinas.

## Synthesis

Reviewing Editor: Matthew Grubb, King’s College London Decisions are customarily a result of the Reviewing Editor and the peer reviewers coming together and discussing their recommendations until a consensus is reached. When revisions are invited, a fact-based synthesis statement explaining their decision and outlining what is needed to prepare a revision will be listed below. The following reviewer(s) agreed to reveal their identity: Joseph Corbo.

Synthesis:

Both Reviewers are very positive about the manuscript, and offer reasonable suggestions for improvement which we all agree should be addressed. One point of clarification which emerged from our consultation discussions together: in response to Reviewer 2’s point 1 about nomenclature, we agree that it is acceptable to refer to the cells as ‘rods’ up front, but with the proviso that the criteria for this designation are explicitly stated. Currently the unstated assumption is that they are fundamentally rods, so please be extremely clear about the basis/bases for this assumption.

Reviewer 1:

The authors of this manuscript investigate the morphology of rod photoreceptors in the simplex retina of the skate. Using serial block electron microscopy 3D reconstruction, they examine the outer segments, inner segments, and synaptic terminals of these photoreceptors with interesting dual scotopic/photopic function. The experiments are carefully done and well described. The results are unexpected and novel, demonstrating that the skate photoreceptor synaptic terminals have a single invagination, as most rods, but also multiple ribbons, as mammalian cones; cone-like telodendria with synaptic vesicles that appear to connect to adjacent rods; and unusually high number of postsynaptic processes invaginating into a single rod terminal. Confirmation of the rod-like structure of the outer segments of skate photoreceptors with separated stacked membranes is also provided.

Overall, these findings are novel and should be of general interest to retinal neurobiologists. They establish further the simplex skate retina as a unique and interesting model system and point to future work on identifying the molecular and cellular mechanisms that enable its single rod photoreceptor type to sustain vision in both scotopic and photopic conditions.

While no major issues are identified in the manuscript, the authors should consider addressing several questions in the Discussion that might provide further insight and provoke the interest of the readers.

- As stated in the Methods, the retinas were dissected in light conditions suggesting that the morphological structures and features identified are present in light-adapted skate rods. While not found in mammals, fish retinas undergo strong diurnal and circadian retinomotor reorganization that involves realignment of rods and cones with respect to the light, as well as a circadian restructuring of the synapses in cones. Thus, while the authors should consider future comparative studies from dark-adapted skate retinas, they might also discuss here the possible effects of light adaptation on the structures that they describe.

- Along the same line, there is no discussion on how the unique morphological properties of skate rod synapses could actually modulate retinal signaling to facilitate both scotopic and photopic vision.

- The authors hypothesize that the hybrid rod-cone architecture of the skate rods is likely to mediate the ability of these cells to function across both scotopic and photopic conditions. While the hybrid synaptic structure and the higher-than-normal connectivity of the rods to second order neurons and to each other could contribute to network adaptation and altered signal processing, previous single-cell suction recordings have also demonstrated that the phototransduction cascade in the outer segments of these cells also undergoes slow light adaptation that shifts the function of these rods from scotopic to photopic conditions. The apparently normal outer segment morphology of the skate rods does not provide insight into the underlying mechanism. The authors should consider discussing this issue and providing their thoughts on the possible phototransduction adaptation mechanisms in skate rods.

- The authors find that, in contrast to the thick outer nuclear layer of the mouse retina, the skate rods are only moderately stacked. This begs the question of how the rod density in the skate retina compares to the rod density in commonly studied amphibian retinas (e.g. salamander), fish retinas (e.g. zebrafish), or mouse retinas. Does the presumably lower rod density in the skate retina bear any relevance for the unusually wide dynamic range of these photoreceptors?

Editorial issues:

- Line 200: the word “location” is repeated twice.

- Line 246: “...number if invaginating processes...” should be “...number of invaginating processes...”

Reviewer 2:

In this study, the authors present 3D EM reconstructions of the retina of the little skate, focusing on the architecture of the photoreceptor cell. The data are for the most part clear and beautiful. This is a welcome contribution, focused on a species belonging to an early-branching class of vertebrates (chondrichthyes) whose retinas are understudied. In general the data support the conclusions. My main reservation is that I don’t agree with the interpretation that the unusual features of the skate’s photoreceptor are indicative of the cell’s hybrid rod/cone nature. This interpretation seems to be largely predicated on comparisons to mammalian rod and cones, which, as the authors themselves state in multiple places, are not necessarily representative of rods and cones in other vertebrate classes. I therefore think that it would be better to recast the results as a novel contribution to our understanding of a poorly studied group of vertebrates which display a single photoreceptor cell type with a variety of striking and unusual ultrastructural features.

I will address the weaknesses of the ‘hybrid’ interpretation in a point-by-point fashion:

(1) The authors refer to the single photoreceptor type of the skate as a ‘rod’ from the very first paragraphs. This nomenclature introduces an unnecessary bias right away. Why not just call it a ‘photoreceptor’? After all, although a prior EM study showed that the membrane disks of the outer segment are non-continuous with the plasma membrane (a typical feature of rods in many vertebrate species), Dowling and Ripps (and Cornwall) previously showed in a long series of elegant studies that the skate photoreceptor behaves physiologically as both a rod and a cone under different light conditions. So, I think it is best to remain agnostic at the beginning of the manuscript as to whether this is in fact a ‘rod’ or a ‘cone’ because the physiology (arguably the most important feature) doesn’t allow one to decide between the two.

(2) The authors report three main findings to support the existence of ‘hybrid’ features in the skate’s photoreceptor cell type: (i) multiple ribbons over a single ‘rod’ invagination’ (ii) multiple invaginating contacts into each ‘rod’ terminal; and (iii) long, abundant telodendria. My contention is that nearly all of these features (or aspects of these features) have been reported to occur in the rods of various non-mammalian vertebrate species. For example, the authors themselves state (lines 230-231) that “In some fishes and amphibians...rods can contain more than one ribbon.”

Furthermore, the authors claim that cones typically have more abundant and longer telodendria than rods. They therefore conclude that the long telodendria of the skate’s photoreceptors are a ‘cone-like’ feature. Once again, I think that this conclusion can only be safely drawn by comparison to a phylogenetically limited set of rods. One can easily find counterexamples in the chapters on retina in Ramón y Cajal’s “Histology of the Nervous System” (English translation by Swanson and Swanson). For example, in Fig. 204 of Vol. II (p. 270) Cajal depicts detailed drawings of the photoreceptors of a ‘passerine bird’. In this figure, the rods (labeled ‘a’) have much more exuberant and longer telodendria than any of the cone types (labeled ‘b’, ‘c’, and ‘d’). My point is that the ‘hybrid’ features of the skate photoreceptor can be found in conventional rods of a variety of vertebrate species, thus undermining the authors’ interpretation.

(3) The authors state (lines 393-394) that they have ultrastructural data on the inner segment mitochondria that also indicate a ‘hybrid’ nature to the skate’s photoreceptor cells, but that they are saving these data for another publication. Why? It really seems that these data ought to be introduced in the present work to strengthen their case.

(4) My main point is that the ‘cone-like’ nature of various structural properties in the skate can only be so designated by reference to a phylogenetically limited selection of ‘rods’. We (as a field) do not yet possess a sufficiently comprehensive phylogenetic sample of photoreceptor ultrastructures to say with any certainty what is truly a rod-like vs. cone-like feature. Therefore it is possible that no universal rod- or cone-defining features even exist. An excellent ‘control’ for the present study would be ultrastructural analysis of the synaptic architecture of rods and cones in closely related elasmobranchs with duplex retinas. Perhaps the synapses of many elasmobranch rods are just like those in the single photoreceptor cell type of the little skate.

Other minor comments:

(1) In lines 7-8, the authors use the phrase “evolutionarily optimized.” What does this mean? Evolution does not usually act to ‘optimize’ function in any clearly definable sense except when that function can be directly compared to some standard of physical optimality. I therefore would discourage use of the term ‘optimized’ in this context.

(2) In line 45, the authors refer to the skate retina as “a naturally occurring cone knock out visual system.” I think it would be better to phrase this differently.

(3) In line 136, “US East Coast” is better written as “the east coast of the United States.”

(4) Lines 157-161 contain a lot of detailed numbers which are important but might be better presented in the Methods to make the main text flow more freely.

(5) In lines 196-197 the authors use the phrase “separated from rod outer membranes”. I think their meaning would be clearer if they reworded it as “separated from the rod plasma membrane.”

(6) Line 200 has an apparent typo “location vertical location”.

(7) In Fig. 3C, I would recommend switching the order of ‘inner segment’ and ‘outer segment’ in the legend to match the left-to-right order of the data in the panels.

(8) The authors claim that Figs. 3B1, B2 confirm the original finding of Szamier and Ripps that the rod outer segment membrane disks are physically non-continous with the plasma membrane. However, I don’t think I can actually see this in Figs. 3B1, B2.

(9) The author refer to the ‘tilt angle’ of the outer segment relative to the inner segment. I have two comments on this. First, the authors should state verbally in the main text what they mean by ‘tilt angle’ (even though they do show it clearly in the figure). Second, the tilt angle typically depends on retinal eccentricity, as shown in the following reference (PMID: 33328242). The authors should therefore make this clearer in the text and also make clear from which retinal eccentricity their sample derives.

(10) In lines 216-218 the authors state “there is little stacking of photoreceptor nuclei in the skate retina, unlike what is often observed in a number of mammalian species.” I find this to be an unclear way of describing what is normally referred to as “ONL thickness”. It has been known at least since the publication of Gordon Walls’ book, “The Vertebrate Eye and Its Adaptive Radiation” in 1942, that nocturnal species (such as the mouse) have thick ONLs with many ‘stacked’ layers of photoreceptor nuclei. In contrast, diurnal species (e.g., diurnal birds) typically have thin ONLs, often consisting of only 1-3 layers of photoreceptor nuclei. Walls depicts this fundamental difference (which obtains across vertebrate classes) in Fig. 72 on p. 177 of his book. Within the order Rodentia one can see both extremes: the nocturnal house mouse with ∼11-12 nuclei-thick ONL vs. the diurnal thirteen-lined ground squirrel with only ∼3-4 nuclei-thick ONL. Thus, I recommend that the authors recast their discussion in terms of ‘ONL thickness’ rather than ‘nuclear stacking’ which is less clear.

(11) Line 376 has an apparent typo “simplex of all”

(12) In regard to the description of ‘hybrid’ photoreceptor states, the authors might wish to cite in their discussion a series of publications from 2005 that all contemporaneously reported the occurrence of a molecularly ‘hybrid’ photoreceptor type in Nr2e3 mutant mouse rods (which were shown to derepress a subset of cone genes, while retaining full rod gene expression): PMID: 15689355, PMID: 16110338, and PMID: 15634773.

## Author Response

Reviewer 1:

1) As stated in the Methods, the retinas were dissected in light conditions suggesting that the morphological structures and features identified are present in light-adapted skate rods. While not found in mammals, fish retinas undergo strong diurnal and circadian retinomotor reorganization that involves realignment of rods and cones with respect to the light, as well as a circadian restructuring of the synapses in cones. Thus, while the authors should consider future comparative studies from dark-adapted skate retinas, they might also discuss here the possible effects of light adaptation on the structures that they describe.

This is a very helpful suggestion and a consideration of the effects of light adaptation on the structures we describe is important. We have added text to the Discussion (lines 468-484) to describe retinomotor movements of rods and cones during diurnal light- and dark-adaptation. Specifically, that rod inner segments elongate bringing outer segments closer to the RPE in the light-adapted state, and that cone inner segments elongate in the dark-adapted state bringing outer segments closer to the RPE, both via a change in the myoid portion of the inner segment of each photoreceptor type. The possible effects of light-adaptation on synaptic vesicle density and synaptic ribbon size has also been added to the Discussion section (lines 466-484).

Finally, to strengthen this point, we have also 1) added references (#8, 24, 83, 84, 85) with additional background on the effects of circadian and diurnal changes on retinal structure and 2) added a statement in the Methods section specifically indicating that retinas were dissected under light-adapted conditions (lines 129-131).

2) Along the same line, there is no discussion on how the unique morphological properties of skate rod synapses could actually modulate retinal signaling to facilitate both scotopic and photopic vision.

Upon careful reflection, we recognize that a discussion of the connection between the structure of skate photoreceptors and their dual function should be included, although it is difficult to frame such a discussion without further functional studies. Nevertheless, even though we do not have physiological evidence for this, we suggest that photoreceptors with different numbers of ribbons may not be equivalent. That is, photoreceptor synaptic terminals with one or two ribbons may be more frequently utilized under scotopic conditions (i.e., rod-like function), while synaptic terminals with three or four ribbons may be utilized more heavily under photopic conditions (i.e., cone-like function). Our structural data (Fig. 4) suggests that the distribution of terminals with different number of ribbons is in a mixed mosaic, rather than a spatial clustering of terminals with specific numbers of ribbons. Furthermore, we think it is possible that ribbon structure and synaptic vesicle density may change differentially during light- and dark-adaptation in photoreceptors with different number of ribbons, as has been described for rods and cones in the goldfish retina (Yazulla and Studholme, 1997; included in revised manuscript as ref# 86). We have included all of the above discussion points in the Discussion section (lines 488-498).

3) The authors hypothesize that the hybrid rod-cone architecture of the skate rods is likely to mediate the ability of these cells to function across both scotopic and photopic conditions. While the hybrid synaptic structure and the higher-than-normal connectivity of the rods to second order neurons and to each other could contribute to network adaptation and altered signal processing, previous single-cell suction recordings have also demonstrated that the phototransduction cascade in the outer segments of these cells also undergoes slow light adaptation that shifts the function of these rods from scotopic to photopic conditions. The apparently normal outer segment morphology of the skate rods does not provide insight into the underlying mechanism. The authors should consider discussing this issue and providing their thoughts on the possible phototransduction adaptation mechanisms in skate rods.

We agree with Reviewer 1’s point in this comment. Light-adaptation in rod outer segments was beautifully demonstrated in single-cell suction recordings done by Cornwall et al., 1989. We have also confirmed outer segment structure findings by Szamier and Ripps, 1983 (i.e., OS membrane discs separated from the plasma membrane; updated Fig. 3 in revised manuscript). To this point, molecular mechanisms of light adaptation could involve key residue changes in skate rhodopsin, as compared to other vertebrate opsins and described by O’Brien et al., 1997 (ref# 9 in revised manuscript). O’Brien and colleagues cloned opsin cDNA from the skate retina and showed that there are residue changes in skate opsin at positions known to interact with phototransduction cascade proteins. For example, a residue change in skate opsin (Gln150Ser), which is considered the site for transducin interaction, might affect transducin activation levels, or even transducin translocation to the inner segment (although, transducin translocation to the IS during light-adaptation has not been demonstrated for skate photoreceptors thus far). This perhaps leads to changes in PDE activation and changes in outer segment cGMP-gated current. In our study, we show that skate photoreceptors have inner segments that are ∼50% longer than outer segments (Fig. 3), which, along with the Gln150Ser substitution in skate rhodopsin, could have an effect on any hypothetical transducin translocation and therefore an effect on the point at which skate photoreceptors switch from high sensitivity to low sensitivity.

We have included the above discussion points, and have included all of them in the Discussion section (lines 592-610).

4) The authors find that, in contrast to the thick outer nuclear layer of the mouse retina, the skate rods are only moderately stacked. This begs the question of how the rod density in the skate retina compares to the rod density in commonly studied amphibian retinas (e.g. salamander), fish retinas (e.g. zebrafish), or mouse retinas. Does the presumably lower rod density in the skate retina bear any relevance for the unusually wide dynamic range of these photoreceptors?

Reviewer 1 brings up an interesting point. We have a good indication of photoreceptor density in the skate retina from the work of Mäthger et al., 2021 (ref# 36in revised manuscript), which uses histological approaches in adult animals to estimate photoreceptor densities of ∼7.7x104 - ∼9.4x104 cells/mm2 in the area of the visual streak (our samples are also from that approximate area). It is worth noting that the eyes of teleost fishes continue to grow throughout life (Kunz, 2007; ref# 99 in revised manuscript) and continue to add new photoreceptors in the periphery. We assume the same is true for skate eyes, as we know from our unpublished observations that they change size as the animal grows. We do not think we can obtain reliable photoreceptor densities from our data, as our EM samples do not cover a large area, but we can compare the photoreceptor densities from Mäthger and colleagues to average photoreceptor densities reported by others in commonly used species. Below are rough approximations of photoreceptor densities in zebrafish, mouse, salamander and xenopus that we found in the literature:

Zebrafish rods ∼ 6.0x104 (Van houcke et al., 2019)

Zebrafish cones ∼ 2.1x104 cells/mm2 (Van houcke et al., 2019; Huckenpahler et al., 2018)

Mouse rods ∼ 3.3x105 - 5.1x105 cells/mm2 (Jeon et al., 1998)

Mouse cones ∼ 8.7x104 - 1.6x105 cells/mm2 (Jeon et al., 1998)

Salamander rods ∼ 4.4x103 cells/mm2 (Zhang and Wu, 2009)

Salamander cones ∼ 3.5x103 cells/mm2 (Zhang and Wu, 2009)

Xenopus rods ∼ 7.3x103 cells/mm2 (Gábriel and Wilhelm, 2001)

Xenopus cones ∼ 6.4x103 cells/mm2 (Gábriel and Wilhelm, 2001)

Note, this is only a rough approximation from the literature and is taken across retinal eccentricities and cone spectral sensitivities, where described. Based on these data, it appears, albeit somewhat surprisingly, that skate photoreceptor density in the visual streak is an order of magnitude higher than what is found for both rods and cones in amphibian retinas. This holds even for photoreceptor densities away from the visual streak, based on values reported by Mäthger et al. (2021). Skate photoreceptors are marginally denser in the visual streak than the total populations of zebrafish rods and cones, but that difference starts to disappear when one moves away from the visual streak and towards the periphery. Unsurprisingly, mouse rods have a density that is an order of magnitude higher, while mouse cones are of similar, or higher density than skate photoreceptors. Thus, skate photoreceptor densities appear to be higher than rod and cone densities in amphibians and teleost fishes, but generally lower than rod densities in mice. This difference could be a function of the difference in photoreceptor and eye sizes between species, and makes it difficult to determine if the density is in some way relevant to the observed dual physiological function in skate photoreceptors.

We have included all of these points in the Discussion section (lines 558-590).

Editorial Comments:

5) Line 200: the word “location” is repeated twice.

Corrected.

6) Line 246: “...number if invaginating processes...” should be “...number of invaginating processes...”

Corrected.

Reviewer 2:

1) The authors refer to the single photoreceptor type of the skate as a ‘rod’ from the very first paragraphs. This nomenclature introduces an unnecessary bias right away. Why not just call it a ‘photoreceptor’? After all, although a prior EM study showed that the membrane disks of the outer segment are non-continuous with the plasma membrane (a typical feature of rods in many vertebrate species), Dowling and Ripps (and Cornwall) previously showed in a long series of elegant studies that the skate photoreceptor behaves physiologically as both a rod and a cone under different light conditions. So, I think it is best to remain agnostic at the beginning of the manuscript as to whether this is in fact a ‘rod’ or a ‘cone’ because the physiology (arguably the most important feature) doesn’t allow one to decide between the two.

Yes, we agree with Reviewer 2’s point here. After some careful consideration, and taking into account both Reviewers’ comments about the “hybrid rod” argument we make here, we thought it best to change the manuscript in a way that remains neutral about the rod or cone identity of skate photoreceptors. Thus, to avoid unnecessary bias and confusion to the potential reader, we have changed the “rod” nomenclature throughout the manuscript with “photoreceptor”. Indeed, the “rod” naming is largely based on outer segment morphology in previous work, and our own work shows anatomical features that make it difficult to shoehorn skate photoreceptors into a single cell identity. To that end, and after carefully thinking about the entirety of Reviewer 2’s comments, we have also decided to change the title of the manuscript to “Ultrastructural characteristics and synaptic connectivity of photoreceptors in the simplex retina of Little skate (Leucoraja erinacea)”, which we hope more accurately informs the reader about the study.

2) The authors report three main findings to support the existence of ‘hybrid’ features in the skate’s photoreceptor cell type: (i) multiple ribbons over a single ‘rod’ invagination’ (ii) multiple invaginating contacts into each ‘rod’ terminal; and (iii) long, abundant telodendria. My contention is that nearly all of these features (or aspects of these features) have been reported to occur in the rods of various non-mammalian vertebrate species. For example, the authors themselves state (lines 230-231) that “In some fishes and amphibians...rods can contain more than one ribbon.” Furthermore, the authors claim that cones typically have more abundant and longer telodendria than rods. They therefore conclude that the long telodendria of the skate’s photoreceptors are a ‘cone-like’ feature. Once again, I think that this conclusion can only be safely drawn by comparison to a phylogenetically limited set of rods. One can easily find counterexamples in the chapters on retina in Ramón y Cajal’s “Histology of the Nervous System” (English translation by Swanson and Swanson). For example, in Fig. 204 of Vol. II (p. 270) Cajal depicts detailed drawings of the photoreceptors of a ‘passerine bird’. In this figure, the rods (labeled ‘a’) have much more exuberant and longer telodendria than any of the cone types (labeled ‘b’, ‘c’, and ‘d’). My point is that the ‘hybrid’ features of the skate photoreceptor can be found in conventional rods of a variety of vertebrate species, thus undermining the authors’ interpretation.

We thank Reviewer 2 for this very helpful comment. As mentioned above, we have carefully considered our claim and we agree with the critique of our “hybrid” interpretation. We especially appreciate bringing up the particular example from Cajal’s work, which escaped our attention previously, but is still very salient today. We have addressed this critique throughout the manuscript (changes highlighted) and have removed, or changed, instances of the “hybrid” interpretation. We have also added text to the Discussion section (lines 620-631) discussing the role of the Nr2e3 nuclear receptor and the associated studies showing a “hybrid” photoreceptor in Nr2e3 KO animals.

Further, we agree that a classification of a photoreceptor as a “hybrid” would not be complete without a more detailed description of said cell’s molecular profile and a robust phylogenetic comparison. Unfortunately, and as Reviewer 2 mention in their general comments, the skate and its close relatives are quite understudied, and, although our laboratory is making some progress, we do not yet have the structural or molecular data to make such phylogenetic comparisons.

3) The authors state (lines 393-394) that they have ultrastructural data on the inner segment mitochondria that also indicate a ‘hybrid’ nature to the skate’s photoreceptor cells, but that they are saving these data for another publication. Why? It really seems that these data ought to be introduced in the present work to strengthen their case.

We have carefully considered this comment and in light of all of Reviewer 2’s comments, and what we agree is an appropriate shift away from the “hybrid” photoreceptor argument, we have decided that our mitochondria data may not make a compelling case. Thus, we have removed the mention of this data in the revised Discussion section (lines 463-465) to avoid any further confusion.

4) My main point is that the ‘cone-like’ nature of various structural properties in the skate can only be so designated by reference to a phylogenetically limited selection of ‘rods’. We (as a field) do not yet possess a sufficiently comprehensive phylogenetic sample of photoreceptor ultrastructures to say with any certainty what is truly a rod-like vs. cone-like feature. Therefore it is possible that no universal rod- or cone-defining features even exist. An excellent ‘control’ for the present study would be ultrastructural analysis of the synaptic architecture of rods and cones in closely related elasmobranchs with duplex retinas. Perhaps the synapses of many elasmobranch rods are just like those in the single photoreceptor cell type of the little skate.

This is another very helpful comment. We agree with Reviewer 2 that ultrastructural data in the literature is not as widely available as molecular or physiological data. Serial structural EM data is even more limited. We are very interested in applying this imaging method to a variety of vertebrate species, including those with simplex retinas, or those that are close elasmobranch relatives of the Little skate. We think a comprehensive comparative approach of photoreceptor structure would be a valuable resource to the field. We have added text to the Discussion section of the revised manuscript (lines 543-556) addressing the point that universal features of rod or cone structure may indeed be difficult to ever find. Several references showing non-traditional photoreceptor structural qualities in different vertebrate species have either been added, or additionally discussed in the context of this comment (please see ref# 82 Morshedian and Fain, 2017; #98 Schott et al., 2016; #96 Walls, 1934).

Other minor comments:

5) In lines 7-8, the authors use the phrase “evolutionarily optimized.” What does this mean? Evolution does not usually act to ‘optimize’ function in any clearly definable sense except when that function can be directly compared to some standard of physical optimality. I therefore would discourage use of the term ‘optimized’ in this context.

We intended this to mean that the skate retina is well-suited for the study of rod circuitry in isolation, as it has only a single photoreceptor cell type, which nonetheless seems to be quite sufficient for scotopic and photopic function and appears an appropriate adaptation to this animal’s ecological niche. However, we recognize how the “evolutionarily optimized” phrasing can be confusing, and perhaps inaccurate here, and we have edited the text to a more general statement about the value of the skate retina in comparative studies (lines 7-10).

6) In line 45, the authors refer to the skate retina as “a naturally occurring cone knock out visual system.” I think it would be better to phrase this differently.

We have rewritten this sentence and removed the “cone-knockout” phrasing as suggested (lines 61-63)

7) In line 136, “US East Coast” is better written as “the east coast of the United States.”

Changed as suggested.

8) Lines 157-161 contain a lot of detailed numbers which are important but might be better presented in the Methods to make the main text flow more freely.

Changed as suggested; same information is already included in the Methods section (lines 154-156).

9) In lines 196-197 the authors use the phrase “separated from rod outer membranes”. I think their meaning would be clearer if they reworded it as “separated from the rod plasma membrane.”

Changed as suggested; “rod” replaced with “photoreceptor”, as discussed in response to comment #1 from Reviewer 2

10) Line 200 has an apparent typo “location vertical location”.

Corrected.

11) In Fig. 3C, I would recommend switching the order of ‘inner segment’ and ‘outer segment’ in the legend to match the left-to-right order of the data in the panels.

Changed as suggested; also, Fig. 3C graph legend changed from “rod inner/outer segment” to “photoreceptor inner/outer segment”.

12) The authors claim that Figs. 3B1, B2 confirm the original finding of Szamier and Ripps that the rod outer segment membrane disks are physically non-continuous with the plasma membrane. However, I don’t think I can actually see this in Figs. 3B1, B2.

Thank you for pointing this issue out. We have replaced panels B1 and B2 in Figure 3 with higher magnification EM images to show the separation of the rod outer segment disks and the plasma membrane.

13) The author refer to the ‘tilt angle’ of the outer segment relative to the inner segment. I have two comments on this. First, the authors should state verbally in the main text what they mean by ‘tilt angle’ (even though they do show it clearly in the figure). Second, the tilt angle typically depends on retinal eccentricity, as shown in the following reference (PMID: 33328242). The authors should therefore make this clearer in the text and also make clear from which retinal eccentricity their sample derives.

To the first point in this comment, we have added an explanation of our “tilt angle” measurement to the main text, as suggested (lines 257-260 and 262-266). Briefly, it is a measure of the angle between a continuous straight line drawn through the middle of a 3D reconstruction of the inner segment (and continuing beyond the structure), and the intersecting straight line drawn through the middle of a 3D reconstruction of the outer segment. 3D angle measurements were performed in Amira software.

To the second point in this comment, we agree that this angle does depend on retinal eccentricity, and we thank Reviewer 2 for pointing us to the beautiful study by Chai et al., 2020. We recognize that we did not include precise information about the retinal eccentricity/location of our samples, and we have included additional text in the Methods section to make this clearer (line 132-136). Our samples derive from the horizontal visual streak area of the retina situated directly above the tapetum lucidum (a reflective strip embedded in the choroid). The tapetum was used as a reference point during dissections. More specifically, samples derive from a few square millimeters taken out of what we assumed was the area of the central horizontal streak, immediately dorsal to the optic nerve head. A similar tilt angle for photoreceptor outer segments was recently shown from histological data in the skate retina by Mäthger and colleagues (PMID: 32840869, and reference #32 in revised manuscript). The tilt angle of the majority of photoreceptors within the horizontal streak they recorded was between 5-25 deg, which we took as a good conformation for the location our samples came from, as the average tilt angle from our reconstructions was ∼16 deg.

14) In lines 216-218 the authors state “there is little stacking of photoreceptor nuclei in the skate retina, unlike what is often observed in a number of mammalian species.” I find this to be an unclear way of describing what is normally referred to as “ONL thickness”. It has been known at least since the publication of Gordon Walls’ book, “The Vertebrate Eye and Its Adaptive Radiation” in 1942, that nocturnal species (such as the mouse) have thick ONLs with many ‘stacked’ layers of photoreceptor nuclei. In contrast, diurnal species (e.g., diurnal birds) typically have thin ONLs, often consisting of only 1-3 layers of photoreceptor nuclei. Walls depicts this fundamental difference (which obtains across vertebrate classes) in Fig. 72 on p. 177 of his book. Within the order Rodentia one can see both extremes: the nocturnal house mouse with ∼11-12 nuclei-thick ONL vs. the diurnal thirteen-lined ground squirrel with only ∼3-4 nuclei-thick ONL. Thus, I recommend that the authors recast their discussion in terms of ‘ONL thickness’ rather than ‘nuclear stacking’ which is less clear.

This is a very helpful comment, and we agree with Reviewer 2 that a description of “nuclear stacking” is less clear than a discussion of ONL thickness. Our description of “nuclear stacking” was perhaps too narrow and a comparison to mouse retina was not appropriate here. In the context of nocturnal and diurnal species, the skate appears to be closer to the anatomy of diurnal species, despite the presence of only a single photoreceptor type.

ONL thickness is only 1-2 nuclei thick in both our histological sections and all of the full cross-section EM data and the nuclei are quite elongated. Photoreceptor nuclei also appear somewhat offset from each other, which prompted our use of the phrase “stacking”. We have changed this phrasing in the main text and have instead discussed ONL thickness (lines 268-275). We have also added an average ONL thickness value we obtained from measurements taken across 3 of our full cross-section EM datasets (avg. thickness ∼ 31.3um)

15) Line 376 has an apparent typo “simplex of all”

Corrected.

16) In regard to the description of ‘hybrid’ photoreceptor states, the authors might wish to cite in their discussion a series of publications from 2005 that all contemporaneously reported the occurrence of a molecularly ‘hybrid’ photoreceptor type in Nr2e3 mutant mouse rods (which were shown to derepress a subset of cone genes, while retaining full rod gene expression): PMID: 15689355, PMID: 16110338, and PMID: 15634773.

Thank you for bringing these studies to our attention. While we have re-examined our “hybrid” interpretation in the revised manuscript, we agree that these publications are relevant to the work in this manuscript and have discussed the similar findings in all of these studies showing that Nr2e3, in concert with Nrl, suppresses expression of cone-specific genes in developing rods (lines 620-641). The appearance of molecularly and, to an extent, structurally “hybrid” photoreceptor in rd7 mice is an intriguing finding. The evolutionarily conserved sequence for Nr2e3, especially in zebrafish, makes it an interesting target to look for in skate retina. Interestingly, however, we have not been able to find Nrl expression in our recently obtained skate retina transcriptome data (these are unpublished observations from a project that is currently in progress).
